# Systematic identification of disease-causing promoter and untranslated region variants in 8040 undiagnosed individuals with rare disease

**DOI:** 10.1186/s13073-025-01464-2

**Published:** 2025-04-14

**Authors:** Alexandra C. Martin-Geary, Alexander J.M. Blakes, Ruebena Dawes, Scott D. Findlay, Jenny Lord, Shan Dong, Susan Walker, Jonathan Talbot-Martin, Nechama Wieder, Elston N. D’Souza, Maria Fernandes, Sarah Hilton, Nayana Lahiri, Christopher Campbell, Sarah Jenkinson, Christian G.E.L. DeGoede, Emily R. Anderson, Toby Candler, Helen Firth, Christopher B. Burge, Stephan J. Sanders, Jamie Ellingford, Diana Baralle, Siddharth Banka, Nicola Whiffin

**Affiliations:** 1 Big Data Institute, University of Oxford, Old Road Campus, Oxford, OX3 7LF UK; 2 Wellcome Centre for Human Genetics, University of Oxford, Oxford, OX3 7BN UK; 3 Manchester Centre for Genomic Medicine, Division of Evolution and Genomic Sciences, School of Biological Sciences, Faculty of Biology, Medicine and Health, University of Manchester, Manchester, M13 9WL UK; 4 Department of Biology, Massachusetts Institute of Technology, Cambridge, MA 02139 USA; 5 School of Human Development and Health, Faculty of Medicine, University of Southampton, Southampton, SO17 1BJ UK; 6 Institute of Developmental and Regenerative Medicine, Department of Paediatrics, University of Oxford, Oxford, OX3 7TY UK; 7 Department of Psychiatry and Behavioral Sciences, UCSF Weill Institute for Neurosciences, University of California, San Francisco, San Francisco, CA 94158 USA; 8 Genomics England, Level 21, One Canada Square, Canada Square, Canary Wharf, London, E14 5AB UK; 9 Department of Bioengineering, Imperial College London, London, UK SW7 2AZ; 10 Manchester Centre for Genomic Medicine, Health Innovation Manchester, Manchester University NHS Foundation Trust, Manchester, M13 9WL UK; 11 Institute of Molecular and Clinical Sciences, St George’s, University of London & St George’s University Hospitals NHS Foundation Trust, London, SW17 0QT UK; 12 Department of Paediatric Neurology, Clinical research Facility, Lancashire Teaching Hospitals NHS Trust, Lancashire, PR2 9HT UK; 13 Manchester Metropolitan University, Manchester, M15 6BH UK; 14 Liverpool Centre for Genomic Medicine, Liverpool Women’s Hospital, Liverpool, L8 7SS UK; 15 Department of Paediatric Endocrinology and Diabetes, Education Centre, Bristol Royal Hospital for Children, Level 6 Upper Maudlin Street, Bristol, BS2 8BJ UK; 16 Clinical Diet and Physical Activity Theme, NIHR Bristol Biomedical Research Centre, University of Bristol, Bristol, BS8 2BN UK; 17 Cambridge University Hospitals, Cambridge, CB2 0QQ UK; 18 Wellcome Sanger Institute, Hinxton, Cambridge, CB10 1SA UK; 19 University of Cambridge, Cambridge, CB2 1TN UK; 20 New York Genome Center, New York, NY NY 10013 USA; 21 Program in Medical and Population Genetics, Broad Institute of MIT and Harvard, Cambridge, MA MA 02142 USA

**Keywords:** Untranslated regions, Promoters, Splicing, Rare disease, Non-coding, Regulatory regions

## Abstract

**Background:**

Both promoters and untranslated regions (UTRs) have critical regulatory roles, yet variants in these regions are largely excluded from clinical genetic testing due to difficulty in interpreting pathogenicity. The extent to which these regions may harbour diagnoses for individuals with rare disease is currently unknown.

**Methods:**

We present a framework for the identification and annotation of potentially deleterious proximal promoter and UTR variants in known dominant disease genes. We use this framework to annotate de novo variants (DNVs) in 8040 undiagnosed individuals in the Genomics England 100,000 genomes project, which were subject to strict region-based filtering, clinical review, and validation studies where possible. In addition, we performed region and variant annotation-based burden testing in 7862 unrelated probands against matched unaffected controls.

**Results:**

We prioritised eleven DNVs and identified an additional variant overlapping one of the eleven. Ten of these twelve variants (82%) are in genes that are a strong match to the individual’s phenotype and six had not previously been identified. Through burden testing, we did not observe a significant enrichment of potentially deleterious promoter and/or UTR variants in individuals with rare disease collectively across any of our region or variant annotations.

**Conclusions:**

Whilst screening promoters and UTRs can uncover additional diagnoses for individuals with rare disease, including these regions in diagnostic pipelines is not likely to dramatically increase diagnostic yield. Nevertheless, we provide a framework to aid identification of these variants.

**Supplementary Information:**

The online version contains supplementary material available at 10.1186/s13073-025-01464-2.

## Background

Current approaches to identify a genetic diagnosis for individuals with rare disease are heavily focused on protein-coding regions of the genome. Even where genome sequencing data are available, analysis methods often exclude variants that are not in or immediately adjacent to protein-coding exons. This is in large part due to the difficulty in interpreting variants outside of these regions, but also due to the increased burden of variant review in a clinical context. Studies that have investigated a wider genomic context have successfully identified variants in non-coding regions that cause penetrant Mendelian disease [1–3]. The majority of these studies have, however, focused on small numbers of individuals, specific phenotypes, and/or limited genetic regions. Consequently, we still do not know what proportion of currently genetically undiagnosed individuals with rare disease have disease-causing variants in non-coding regions.

In this work, we focus on promoters and untranslated regions (UTRs) given that these regions can be confidently linked to known disease genes, and variants within them can significantly disrupt normal gene regulation and have previously been implicated in rare disease [4, 5]. In short, they provide the best opportunity to expand clinical screening into non-coding regions.

UTRs are regulatory sequences encoded immediately up- and downstream of the coding sequence (CDS) of protein-coding genes. These regions have important roles in regulating RNA stability, RNA localisation, and the rate of CDS translation [6, 7]. Variants in UTRs that disrupt these regulatory processes have been shown to cause rare disease through a variety of mechanisms [8]. For example, 5′UTR variants that disrupt translation by creating upstream start codons (uAUGs) or perturbing upstream open reading frames (uORFs) cause a range of phenotypes including in genes causing severe developmental disorders (e.g. *NF1* [9] and *MEF2C* [2], whilst variants directly upstream of the CDS in the *GATA4* gene, that alter the ‘Kozak’ consensus (i.e. the AUG start codon and surrounding motif) have been linked to atrial septal defect [10]. Variants resulting in aberrant splicing of the *PAX6* 5′UTR are a frequent cause of aniridia [11]. 3′UTR variants that disrupt polyadenylation signals or RNA Binding protein (RBP) binding sites in the α and β-globin genes have been found in individuals with α and β-thalassemia [12]. A comprehensive search for 5′UTR variants in retinal disease patients uncovered variants that cause disease through a variety of mechanisms [13].

Proximal promoters comprise an open region of chromatin spanning both up- and down-stream of the transcription start site (TSS) to which transcription factors and polymerase bind to initiate transcription. Variants within promoter regions that disrupt transcription by altering transcription factor binding, or by changing methylation patterns have been identified as being causal of a number of diseases, including *TERT* promoter variants in idiopathic pulmonary fibrosis [14] and *CAMK1D* promoter variants in type 2 diabetes [15]. Whilst there are many documented mechanisms through which UTR and promoter variants cause rare phenotypes, our knowledge is likely far from complete. It is also unclear to what extent increasing our understanding of, and regularly including these regions in clinical testing pipelines, will uncover novel diagnoses for currently undiagnosed individuals with rare disorders.

Here, we used the Genomics England 100,000 Genomes Project (GEL) dataset to systematically identify and annotate variants in promoters, UTRs, and UTR introns in 8040 undiagnosed trios. We developed a reproducible annotation approach with high specificity that can be used in clinical settings without dramatically increasing the number of candidate variants for manual review. After employing strict region-based filters, we identified ten likely diagnostic variants, nine de novo and one additional overlapping variant. Comparing individuals with rare disorders to matched controls, we did not identify a significant burden of rare potentially disruptive variants collectively across any region type or variant annotation, although this may be due to limited statistical power. Our results highlight how promoter and UTR regions can be effectively searched for new diagnoses in rare disease patients and we outline a framework for identification and annotation of such variants in large-scale cohorts.

## Methods

### Identifying known disease genes using PanelApp

PanelApp gene panels were obtained from panelapp.genomicsengland.co.uk using lynx v2.8.9 [16], on 12/09/2022. These were filtered to include only the 6504 genes where the strength of association for one or more gene panel was ‘green,’ corresponding to those with a confident link to the phenotype.

We further filtered to only include genes known to cause a disorder with a dominant mode of inheritance (MOI), inclusive of any genes associated with both dominant and recessive phenotypes. Finally, we selected only genes with transcripts in the MANE v1.0 dataset [17]. In total, we included 1536 genes.

### Annotating non-coding regions of interest

Transcripts were defined using MANE v1.0, inclusive of 19,062 MANE ‘select’ and 58 ‘plus clinical’ transcripts [17]. One thousand five hundred and sixty seven transcripts corresponded to the 1536 known disease genes identified above.

UTR exon and intron coordinates were taken directly from the MANE .gff file.

Proximal promoter regions were defined using candidate *cis* regulatory elements (cCREs) overlapping the TSS of each MANE transcript obtained from ENCODE [18]. Specifically, these incorporate chromatin modification and DNA accessibility data across 706 human biosamples spanning 369 tissues. Accurate promoter definition is hampered by their tissue specificity. In tissues where a promoter is inactive, it is often marked by a minimal nucleosome free region, but this region may be expanded when the promoter is active. To account for this, as well as promoters that are not annotated at all in the ENCODE dataset, we calculated the average size of all ‘promoter-like’ cCREs that overlap with TSS of MANE transcripts. We calculated the 25th and 75th centiles of the distribution of distances these cCREs extend up- (25%=181 bp; 75%=266 bp) and downstream (25%=67 bp; 75%=139 bp) of the TSS (Additional file 2:Fig S1). The 25th centiles (−181 bp to +67 bp from TSS) and 75th centiles (−266bp to +139bp) were used to define a ‘minimal’ and ‘maximal’ promoter region respectively.

For transcripts with a cCRE that overlaps the TSS:

- If the cCRE extends ≥ 181 bp upstream and ≥ 67 bp downstream of the TSS (i.e. at least the minimal 25th centile definition), the exact region defined by the cCRE is used (Additional file 2:Fig S1d; *n*=7368).
- If the cCRE falls short in either (or both) direction(s), it is extended to reach the 25th centile distance in that/those direction(s) (Additional file 2:Fig S1e; extended upstream *n*=2953; extended downstream *n*=2918, extended in both directions *n*=464).

For transcripts with no TSS overlapping cCRE (*n*=5417), the 75th centiles are used to define a promoter region that stretches 266 bp up- and 139 bp down-stream of the TSS.

We used bedtools [19] to exclude any positions from our defined UTR, UTR intron, and promoter regions that intersect with a CDS position in any MANE transcript. In total, we defined 20,417,669 promoter and UTR bases across the 1567 green PanelApp genes, for an average of 13,030 bases per transcript (min=264, max=791,387), and between 17 and 18,786 bases per region (Additional file 1:Table S1). The final set of promoter and UTR regions defined across all green PanelApp genes is in Additional file 2:Table S2.

### Cohort details

We accessed genomic and phenotypic data from GEL v15 [20] part of the National Genomics Research Library. The data used in this study were collected from 75,856 rare disease participants recruited via participating genomics ‘hubs’ within the United Kingdom’s National health service. The de novo variant data [21] was derived from a set of 12,572 families (13,913 trios) from within this larger cohort. The aggregated variant data [22] data set comprises the combined genomic variant call data from a subset of 78,143 participants within the main cohort from GEL v9 [23].

Analyses within the de novo section of this manuscript were performed on samples from the 13,913 trios; burden testing was performed using samples from the 78,143 participants included in the aggregated variant data.

### Identifying and filtering de novo variants

We used a dataset of previously identified and filtered high confidence de novo variants (DNVs) within GEL [21], accessed using the GEL RLabKey API [24]. These DNVs were identified from trios for which parentage had been genetically confirmed. We filtered individuals to remove any with subsequently withdrawn consent, and to only include those with a ‘participant type’ of ‘proband’, where neither parent was classified as ‘affected’ or had any associated Human Phenotype Ontology (HPO) terms, and for whom variant calls were on the GRCh38 reference genome. Finally, we excluded participants with an identified coding diagnosis (see below). This resulted in a set of 8040 trio probands (Additional file 2:Fig S2).

Variants were filtered to only those that passed the most stringent set of GEL filters [25]. We removed variants with allele frequency (AF) ≥ 0.00005 or allele count (AC) ≥5 in the GEL defined set of 55,603 unrelated individuals or with AF ≥ 0.00005 in any of the major population groups in gnomAD v3.1.1 [26]. We restricted our analyses to DNVs within our defined promoter and UTR regions of PanelApp genes with high-confidence phenotypic associations (flagged as ‘green’ genes) for the individual’s phenotype. Of note, 309 probands did not have any assigned green dominant genes. This resulted in a set of 1311 DNVs, in 1118 probands.

### Identifying individuals with existing diagnoses

A list of all participants for whom a confirmed diagnosis was recorded was obtained from the Genomics England ‘exit questionnaire’ table, identified as those for whom the family case was flagged as “solved”. The proposed disease-causing variant in each case was then cross referenced with MANE V1.0 coding sequence coordinates, with variants mapped onto GRCh38 using the ‘LiftOver’ [27] tool where required. Any individual found to have a diagnostic variant in a protein-coding position was excluded from the analysis (*n*=1625; 16.8%). Participants with diagnostic variants that fall outside of coding regions were retained as positive controls for use in the later validation of our method.

### Region-level variant annotations

Variants were annotated using Ensembl’s variant effect predictor (VEP) v99.1 [28] with UTRannotator [29], SpliceAI v1.3 [30], and CADD v.1.6 plugins [31], as well as custom annotations for PhyloP 100 way vertebrate conservation scores [32], and ClinVar [33] clinical significance annotations (accessed 2022/08/12). Variants were excluded if they were reported in ClinVar as ‘benign’, ‘likely benign’, ‘benign/likely benign’, or ‘protective’.

**Fig 1:**
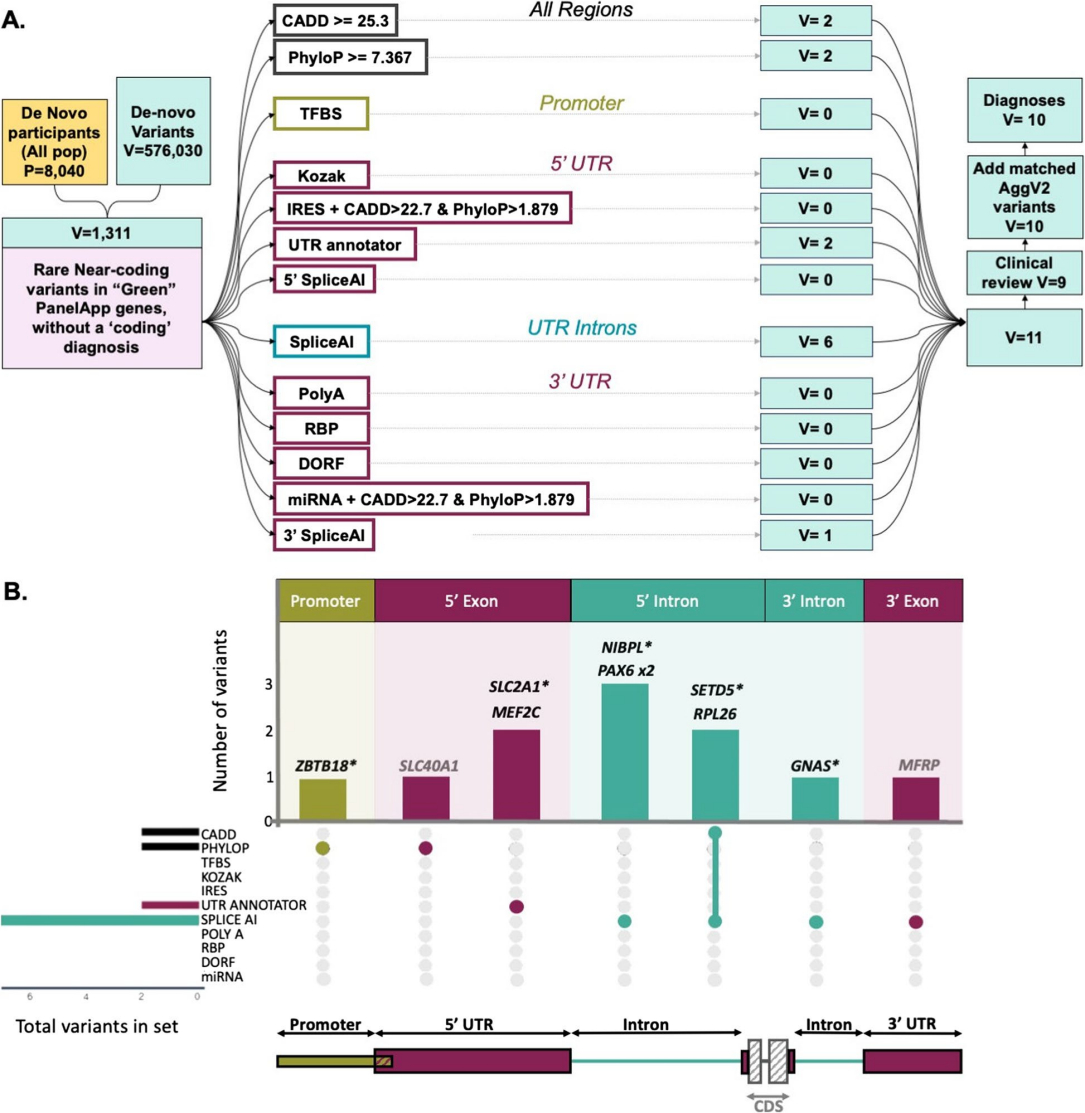
Prioritised de novo variants split by region and variant annotations. DNVs were identified from the Genomics England de novo dataset in the following regions: Promoter (mustard), UTR exons (raspberry), and UTR introns (teal). **A** Flowchart showing de novo variant counts for all steps in our pipeline and the annotations used to prioritise variants in each region type. Filtering steps are shown in pink boxes. Initial participants without a diagnosis attributed to a coding variant are shown in gold box. De novo variant counts of each stage are shown in pale green boxes. **B** Upset plot showing genes with variants prioritised by our pipeline. The gene names corresponding to identified DNVs are written above the corresponding bar. Those in black represent likely diagnoses (nine probands), with those in grey not being a good phenotypic match (two probands). Novel potential diagnoses are marked by an asterisk. Vertical bars in the top panel denote the number of variants identified with specific region and variant annotations that are represented by the bar colour (region annotations), and in the upset plot below (variant annotations). The total number of DNVs with each variant annotation is shown by the horizontal bars to the left of the upset.

The datasets and annotations used for variant annotation are listed in Additional file 2:Table S3 and a schematic of the approach used is shown in Figure 1A.

Variants across all regions were annotated with CADD PHRED [31] and PhyloP [32] scores. For variants with multiple recorded scores, the maximum was taken. We note that thresholds for these scores have been calibrated only for missense variants [34], but no alternative exists for non-coding region variants due to the paucity of variants available to benchmark against. Due to this, CADD PHRED and PhyloP scores were not used to prioritise variants in deep intronic regions (>20 bp from the end of the exon), to reduce noise. In all other regions, variants with CADD ≥25.3 or PhyloP ≥7.367 were prioritised (‘supporting’ evidence thresholds towards pathogenicity taken from Pejaver et al. [34]).

#### Annotating variants impacting splicing

For SpliceAI scores, we took the highest delta value across all predictions. For the de novo variant analysis we set a threshold of 0.2, which is the recommended cutoff for high sensitivity, to avoid excluding potentially diagnostic variants. For the burden testing analysis, we used a cutoff of 0.5 as a trade-off between high sensitivity and high specificity that is more appropriate for burden testing. This is the threshold recommended by the authors of SpliceAI for ‘general purposes’ [30]..

#### Annotating UTR variants impacting translational regulation, miRNA-binding sites, and RNA-binding protein binding sites

5′UTR variants annotated by UTRannotator [29] were filtered to identify those with the highest likelihood of disrupting translation. To this end, we extracted all variants annotated as:

- uAUG gain resulting in creation of an overlapping open reading frame (oORF) with a strong or moderate Kozak consensus sequence;
- uSTOP loss, with no alternate stop prior to the CDS start (i.e. also resulting in an oORF) and a strong or moderate Kozak consensus sequence;
- uAUG loss, with strong Kozak consensus sequence;
- uFrameshift resulting in an oORF with a strong or moderate Kozak consensus sequence.

Internal ribosome entry site (IRES) data were obtained from IRESbase [35] on 23/08/2022, microRNA (miRNA) binding sites were obtained from the literature [36–39], and downstream open reading frame (dORF) coordinates were obtained from Chothani et al. [40]. For each, locations were cross referenced with our variant positions, and any intersecting variant flagged. Given the large proportion of variants that fall into miRNA and IRES sites, we excluded any variants that also had a CADD ≤ 22.7 or PhyloP ≤ 1.879. These scores were suggested by Pejaver et al. as supporting evidence for a benign classification [34].

Kozak consensus sequence variants in the -3 position were identified with reference to MANE v1.0 CDS start positions (i.e. the R of the gccRccAUGG motif). Any variant that changed a reference A or G to a C or T was annotated as potentially Kozak disrupting [41].

RNA binding protein predictions were generated using the methods detailed in Findlay et al. [42] for all possible variants within motifs that are proximal to ENCODE eCLIP sites that are also high affinity sites as predicted by RBPamp [43]. These were intersected with our variants and filtered to retain only those with a reference affinity of ≥ 0.1 and with an impact of ‘loss of binding’ predicted by the RBP binding affinity model (defined as alternative allele affinity / reference allele affinity < ${}^{1}\!\left/ \!{}_{3}\right.$).

#### Annotating polyadenylation signal motifs

Using MANE [17] v1.0 mRNA sequences, we identified the locations of all 3′UTR AAUAAA and AUUAAA polyA signal motifs. These were then cross referenced with primary polyA sites (those with the highest mean TPM from polyA DB [44]), and the closest upstream motif within 30 bps was retained. Comparing these data with that of motifs identified by Shiferaw et al. [45], we find that 80.5% are concordant. It is likely that unmatched motifs are a result of differences in polyA cleavage site selection, alongside regional differences caused by transcript vs gene-level annotation [46].

#### Identifying transcription factor binding sites

Transcription factor binding site (TFBS) locations were obtained from ENCODE [47] and converted using bigBedToBed [48] on the command line, resulting in 4,465,728 TFBS footprints. Any variant not within a footprint identified by ENCODE as falling within the ‘core’ region of a DNase I hypersensitive (DHS) peak was excluded. Remaining variants were annotated using FABIAN [49], limiting to only transcription factor flexible models as these have been shown to outperform positional weight matrices [50]. The resultant data was transformed to produce one score per transcription factor, per variant:

#### Score = (ΣA1:AN)/N

Where *A* is each model’s predicted change in binding affinity and ‘*N*’ is the total number of these predictions provided for that transcription factor. Scores ≥ 0.04 were recorded as predicted gain and those ≤ −0.04 as predicted loss. For each variant, we then calculated the mean gain/loss/total scores and retained any variant with a loss score ≤ −0.4.

### Clinical review of candidate variants

For each participant with a candidate diagnostic de novo variant, we compared the similarity between the HPO terms assigned at recruitment with the phenotype expected for a heterozygous variant in the gene. Given that all of the genes in which we identified candidate variants had been linked to a loss of function mechanism, variants were interpreted under the assumption that they caused loss-of-function (LoF) and were of high penetrance. Expected phenotypes for each gene were sought from OMIM [51] and the published literature. Where we identified a plausible phenotype match, we raised a clinical collaboration request with Genomics England via the secure ‘Airlock’ protocol [52] to contact the recruiting clinician.

### Assessing performance using ClinVar

Using ClinVar downloaded on 2023/08/19 (*n*=1,380,750 variants), we identified variants overlapping one of our annotated regions (*n*=55,081 unique variants). We filtered to high confidence pathogenic/likely pathogenic variants, defined as those classified as ‘Pathogenic’, ‘Likely_pathogenic’, or ‘Pathogenic/Likely_pathogenic’, and reviewed as ‘reviewed_by_expert_panel’, ‘criteria_provided,_multiple_submitters,_no_conflicts’, or ‘practice_guideline’ (*N*=123). We compared these to variants annotated as ‘Benign’ or ‘Benign/Likely_benign’ (*N*=3364). Each set of variants was then annotated and filtered as detailed above for de novo variants, with filtering on allele frequency only using gnomAD.

### Defining case and control sets for burden testing

From GEL version 15 [20], we selected participants meeting all of the following criteria:

1. Variants called on genome build ‘GRCh38 and with delivery version ‘V4’
2. Consent not subsequently withdrawn
3. Karyotype one of ‘XX’, ‘XY’, ‘NA’, ‘Other’ and karyotypic and phenotypic sex not in conflict

Cases were defined as:

1. Individuals with a participant type of ‘proband’
2. With at least one ‘green’ PanelApp gene in a virtual gene panel assigned to them
3. Without an existing coding diagnosis (see above).

Controls were taken as the unaffected parents of participants with rare disease. Defined as:

1. Participant type is ‘Mother’ or ‘Father’
2. Affected status is ‘Unaffected’
3. No recorded HPO terms

The genetically inferred ancestry of each participant, as calculated by GEL, was obtained from LabKey. Participants with a single origin ancestry match of 99% or greater were retained and defined as that ancestry [53]. Through this approach, we defined a total of 19,220 cases and 20,683 controls (Additional file 2:Fig S3).

### Filtering aggregated variants

Variants within MANEv1.0 transcripts, for all potential case and control participants that passed all internal QC filters were extracted from the aggregated variant VCF files in GEL [54].

In line with recommendations from Pedersen et al. [55] we filtered variants to those with genotype quality (GQ) ≥ 20, read depth (DP) ≥ 10, missingness ≤ 5% heterozygous allele balance (AB) 0.2 ≤ AB ≤ 0.8, and homozygous AB ≤ 0.02. If a variant call failed one or more of these filters in ≥ 25% of cases, that call was excluded. We further filtered to only those with GEL internal and gnomAD (v3.1.1; in any population) AF ≤ 0.0001. We retained a total of 18,498,584 variants, a mean variant count per individual of 463.59 (461.74 in cases and 465.74 in controls).

### Participant matching

To exclude any individuals with very high numbers of called variants (suggestive of systematic error), we calculated a population-specific *Z*-score per participant as follows:

z = (x-μ)/σ

Where ‘*x*’ is the variant count in that participant, across all MANE transcripts, from the start of the promoter to the transcript end, ‘*μ*’ is the population mean, and ‘*σ*’ is the population standard deviation, where the population is all individuals defined as being of the same genetic ancestry (see above). Participants with a *Z*-score of ± 2 were dropped (*N*=1560; 728 probands, 832 controls) resulting in a set of 18,492 probands and 19,851 control participants.

Within each cohort, we removed individuals with KING scores [56] ≥ 0.0442 within the Genomics England relatedness data [53], indicative of being a 3rd degree relative, by randomly selecting one participant for removal in an iterative process until no further relatedness in individuals was detected.

We then matched each proband 1:1 with a single control participant by sex and genetically inferred ancestry, ensuring that the matched proband and control did not share a family ID. The resultant matched cohort consisted of 18,304 probands, paired with 11,641 unique controls (some controls were matched to more than one proband). To avoid potential biases when matching participants caused by low population numbers, we limited to genetically inferred ancestries where the number of both case and control participants was > 200. This resulted in a cohort of 17,641 case probands, and 11,227 control participants with either European or South Asian genetically inferred ancestry (Additional file 2:Table S4).

Given the disparity in gene panel size, with many probands having over 500 assigned green dominant genes (Additional file 2:Fig S4), to reduce noise we filtered probands to include only those to whom 100 or fewer green dominant PanelApp genes had been assigned (28% of all probands). This resulted in a final cohort of 7862 probands and 6371 matched controls.

### Burden testing

Aggregated variants filtered as above were further restricted to those with AF ≤ 0.00005 for both internal and gnomAD major population frequencies and to exclude any with an allele count (AC) across the entirety of AggV2 of ≥ 5. These 1,079,616 variants were annotated and filtered with reference to the annotations described above (Additional file 2:Fig S5).

A simple burden test was performed across all defined promoter and UTR regions and variant annotations comparing individuals that had one or more annotated variants meeting our criteria in any promoter or UTR region to those that did not, using one-sided Fisher’s exact test, to test whether cases were enriched for prioritised variants compared to controls (Additional file 2:Table S5). The test was repeated for each region annotation separately. A Bonferroni adjusted *P*-value threshold of ≤0.0031, correcting for 16 tests was used to assess statistical significance.

Power estimation was performed using the statmod library’s power.fisher.test [57] function in R, with a one-sided hypothesis of ‘greater’, alpha 0.0031, 1000 replicates, and seed set to 42.

To estimate the number of participants required to see a significant enrichment across all region and variant annotations, we iteratively increased the number of case and control participants by 1, whilst maintaining the proportion of observed cases and controls with candidate variants. Fisher’s tests were performed for each iteration, until the resulting *P*-value was ≤0.0031.

### Analysing de novo variants in autism

We annotated de novo variants identified in 4199 trios (2317 case, 1882 control) from the Simons Foundation Autism Research Initiative (SFARI) Simplex Collection (SSC) [58]. We identified 1678 variants (920 in cases, 766 in controls) in annotated promoter and UTR regions of 664 NDD associated genes (FDR ≤ 0.05) from Fu et al. [59]. Variants were annotated as detailed above for de novo variants.

### RNA sequencing and DNA methylation analyses

Blood was collected from a subset of 100,000 Genomes Project probands in PaxGene tubes to preserve RNA at the time of recruitment. RNA was extracted, depleted of globin and ribosomal RNAs, and subjected to sequencing by Illumina using 100-bp paired-end reads, with a mean of 102M mapped reads per individual. Alignment was performed using Illumina’s DRAGEN pipeline. IGV [60] was used to inspect sequencing reads and generate Sashimi plots to show splicing junctions supported by 5 or more reads in areas of interest. FRASER2 [61] and OUTRIDER [62] were used to detect abnormal splicing events and expression differences with 499 samples used as controls.

DNA methylation array testing was performed on a diagnostic basis as described previously [63, 64].

Unless otherwise stated, all analyses were performed using R Statistical software version 4.0.2 [65], with the packages ‘dplyr’ [66], ‘tidyr’ [67], ‘stringr’ [68], ’Rlabkey’ [24], ’UpSetR’ [69], and ‘ggplot2’ [70].

## Results

### Strict region-specific filtering prioritises likely deleterious de novo promoter and UTR variants

We identified 685,438 rare (AF ≤ 0.005%) high-confidence DNVs in 9665 trio probands with unaffected parents in GEL (70.9 per proband). Eight thousand and forty of these probands (83.2%) did not have an existing confirmed diagnosis attributed to a variant in a protein-coding region. We filtered to include only DNVs in UTR exons and introns (both defined using MANEv1.0 transcripts) and promoter regions (defined by ENCODE candidate *cis* regulatory elements; see Methods). We limited our analysis to variants which fell in or near known monogenic disorder genes (3316 variants) and filtered these to genes which could be plausibly associated with the participant’s phenotype. Accordingly, we filtered for DNVs of genes flagged as ‘green’ in one or more PanelApp [71] gene panel(s) assigned to the individual, and which were associated with disorders with a dominant mode of inheritance. In total, we proceeded with 1311 candidate DNVs in 1118 probands.

To identify likely disease-causing DNVs, we used a region-specific annotation and filtering approach (Figure 1A). We prioritised 5′UTR variants that create uAUGs or disrupt uORFs using UTRannotator [29], that overlap *IRES* defined by IRESbase [35], or that lead to disruption of the Kozak consensus sequence [41]. 3′UTR variants were prioritised if they disrupt a polyadenylation site or signal sequence, disrupt a miRNA binding site, disrupt an RBP motif, or if they disrupt the start/stop of a dORF with evidence of translation from ribosome profiling (from Chothani et al [40]). Given the large numbers of variants annotated as within IRES or miRNA binding sites, these variants were further filtered to remove any with CADD (<22.7) or PhyloP (<1.879) scores in support of being benign [34]. Across all UTR exons, and in 5′ and 3′ UTR introns, variants with SpliceAI masked delta scores ≥ 0.2 were prioritised. Promoter variants were prioritised if they are predicted to disrupt a transcription factor binding site using FABIAN [49]. Finally, across all regions, variants with a CADD score ≥ 25.3 and/or a PhyloP score ≥ 7.367 were prioritised, irrespective of whether they had any other annotation (thresholds equivalent to ‘supporting’ evidence towards pathogenicity taken from Pejaver et al. [34]). After filtering to only include variants with one or more of these annotations, we retained eleven candidate DNVs (0.8% of the initial 1311 DNVs) each found in a different individual (Figure 1B; Additional file 1:Table S1).

### Promoter and UTR DNVs provide a diagnosis for undiagnosed individuals with rare disease

Of the eleven remaining candidate variants, nine (82%) were assessed to be a good match for the individual’s phenotype after detailed clinical review (see ‘ Methods’). Three of these had been flagged as diagnostic variants in GEL (in the ‘exit questionnaire’ table) prior to starting this work: two 5′UTR splicing variants in *PAX6* in two individuals with aniridia (OMIM:617141) and one 5′UTR splicing variant in *RPL26* in an individual with a previously undiagnosed monogenic disorder. A further variant, a 5′UTR variant that creates an upstream start codon in *MEF2C*, we previously identified as occurring de novo in three unrelated individuals with severe developmental disorders [2]. Our approach successfully prioritised all rare DNVs within our candidate regions that had previously been identified as likely diagnostic in GEL. Together, these data demonstrate that our pipeline effectively identifies known diagnostic variants.

**Fig 2:**
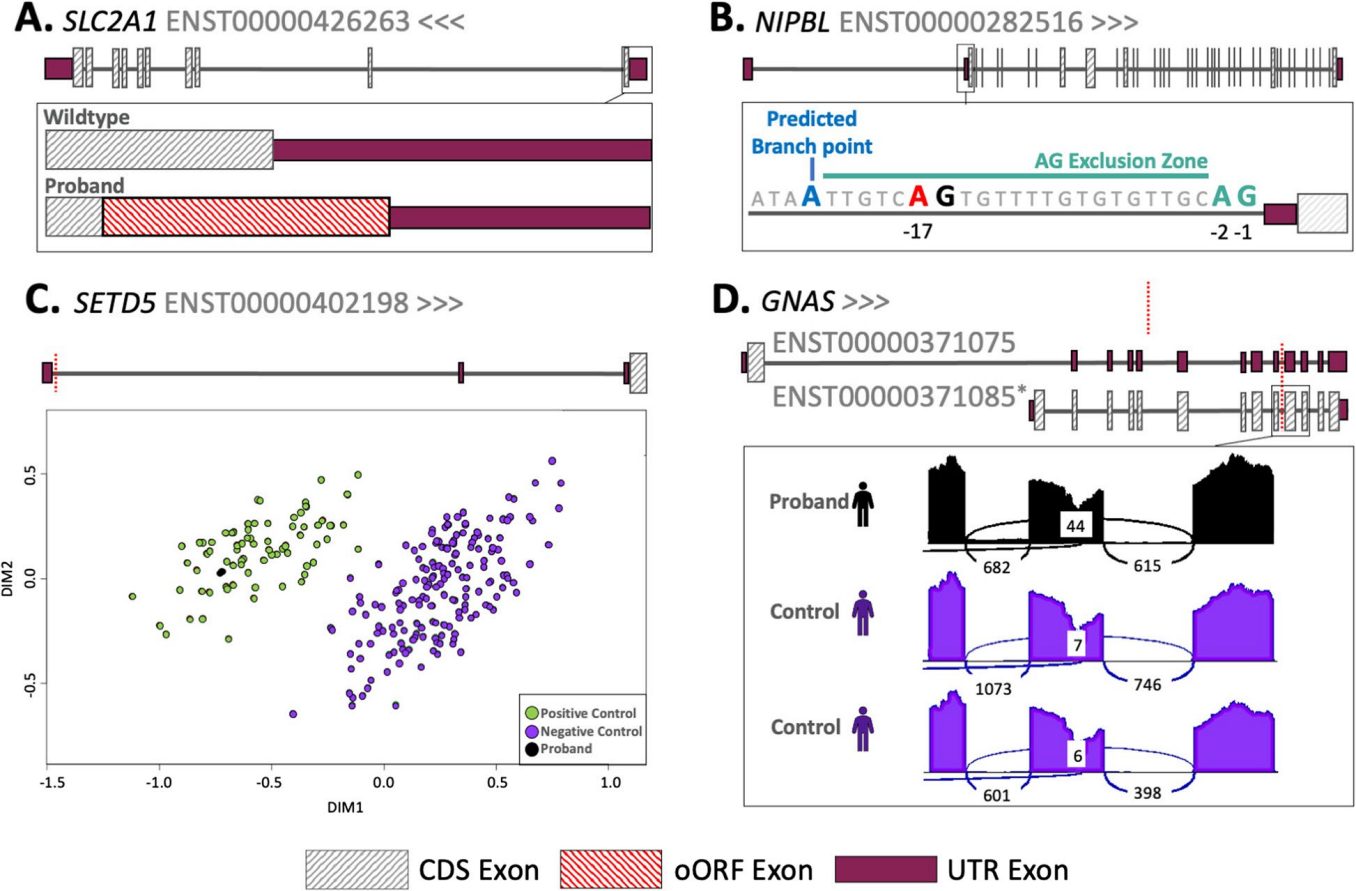
Candidate diagnostic de novo variants. **A** Gene diagram showing the creation of an out of frame overlapping ORF (oORF; in red) in the *SLC2A1* gene in the proband. **B** Illustration of the AG exclusion zone in the *NIPBL* gene. The T>A variant at the -17 position is marked in red, the most strongly predicted branch point (Branchpointer [73] 0.48), directly upstream of the AG exclusion zone is shown in blue. **C** Multidimensional scaling plot showing differential methylation in *SETD5.* The position of both variants found in this gene is shown as red dotted lines. **D** Sashimi plot showing aberrant splicing in the MANE Plus clinical transcript ENST00000371085. The proband shows some retention of the intron containing the variant (which is marked by a red dotted line) and increased skipping of the following exon compared to the controls (6.06% vs 0.65% and 1%)

Four of the remaining five variants represent likely new diagnoses: (1) a 5′UTR uAUG-creating variant in *SLC2A1* in a patient with GLUT1 deficiency syndrome (OMIM:606777) that was not flagged by GEL as diagnostic, but that has been published previously [3] (Figure 2A). This uAUG is created into a strong start codon context and functional studies support its translation [3]. Translation from this uAUG will prevent translation of the downstream CDS, leading to loss-of-function (Figure 2A). After returning this diagnosis to the recruiting clinical team, it was classified as Likely Pathogenic and the individual is now on treatment; (2) A *NIPBL* splice disrupting (SpliceAI=0.24) variant 17 bp upstream of the final 5′UTR acceptor site in a participant with a phenotype closely related to Cornelia de Lange syndrome (OMIM:122470, Figure 2B). This variant introduces an AG dinucleotide which is predicted to result in a premature acceptor; however, the positioning of this within the ‘AG exclusion zone’ may also cause skipping of the exon containing the CDS start codon or other splice defects [72] (Figure 2B). The exact impact of this variant will need to be confirmed through RNA studies, but RNA was not available for the patient; (3) A promoter variant that is located in a highly evolutionarily conserved position (PhyloP=7.426) 13 bp upstream of the TSS of *ZBTB18* in a participant with Intellectual disability; (4) A 5′UTR splice-site variant in *SETD5* in an individual originally suspected to have Silver Russell Syndrome (OMIM:180860). This variant is predicted to result in loss of the splice donor (SpliceAI=0.97) of the first 5′UTR exon at the canonical +1 position. DNA methylation signature analysis in this patient revealed an episignature consistent with *SETD5-related neurodevelopmental disorder* (Figure 2C) and no other candidate variants were identified after screening the protein-coding regions of *SETD5*.

We also prioritised a cryptic splice variant in *GNAS* (SpliceAI=0.67) in a participant with hypothyroidism. Whilst we originally annotated this variant as within a 3′UTR intron for the MANE Plus Clinical transcript, the intron is between two CDS exons of the MANE Select transcript. Blood RNA-sequencing from the patient showed evidence of abnormal splicing of the MANE Select transcript, including intron retention (FRASER2 adjusted *P*=1.67×10^−23^), and significantly reduced expression (OUTRIDER adjusted *P*=0.0019; fold-change=0.66; Figure 2D); however, the exact mechanism through which this variant could lead to disease is unclear.

For all candidate variants, we checked whether they were found in any other individuals across the full GEL cohort (i.e. not limiting to full trios or DNVs). Whilst we did not observe recurrence of any of the exact variants identified, we did identify a second participant with a different *SETD5* variant at the same genomic position (chr3:9397974 CAAGGT>C, hg38). On closer investigation, this variant is consistent with a germline de novo, but it fell just below the required coverage in one parent so it was excluded from the conservative high confidence de novo callset. DNA methylation signature analysis also confirmed *SETD5* as the diagnosis in this individual (Figure 2C). In total, we identified a likely disease-causing promoter and UTR DNV in ten of 8040 individuals (0.0012%; nine initially prioritised variants and one additional *SETD5* variant) who did not previously have a coding diagnosis. We classified all six newly identified variants as Likely Pathogenic following the ACMG/AMP guidelines (Additional file 2:Table S6) [8, 74].

### Our approach shows high accuracy when tested using ClinVar

To further test the utility of our approach to identify known pathogenic and benign variants, we used ClinVar. We identified 3364 (likely) benign, and 123 (likely) pathogenic variants in our defined promoter and UTR regions across all genes (Table 1). Following annotation, 66/123 (53.7%) pathogenic variants, and only 24/3364 (0.71%; Fisher’s *P*-value = 2.59×10^−84^) benign variants were prioritised by our pipeline (Additional file 2:Tables S7 & S8). Of the 57 pathogenic variants that were not prioritised, 15 (26.3%) were annotated as too common (allele frequency >0.00005 in gnomAD), but all 15 were in genes primarily associated with recessive disease (*HBB* (x8), *GJB2* (x3), *MYO7A*, *RAPSN*, *HBA2*, and *CHRNE*). Four variants (7.0%) were not prioritised when using precomputed SpliceAI scores provided by VEP, but would have been prioritised if SpliceAI was run directly with up-to-date transcript models. The majority of the remaining variants (24/38; 63.2%) were annotated solely as promoter variants, highlighting limitations of our pipeline in annotating variants in these regions (Additional file 2:Table S8). Conversely, the majority of the benign variants that were aberrantly prioritised (18/25; 72.0%) had SpliceAI scores ≥ 0.2.

**Table 1:** Counts of prioritised and not prioritised ClinVar variants. Variants are annotated with classification and region information. Variants annotated as overlapping the 5′UTR and promoter are included only in the 5′UTR counts. Two un-prioritised benign variants that overlap both 3′ and 5′ UTR exons are omitted from the region specific counts, but included in the total for that column

**Region**	**Prioritised pathogenic (%)**	**Not prioritised pathogenic**	**Prioritised benign (%)**	**Not prioritised benign**
Promoter	17 (36.2%)	30	0 (0%)	376
5′ UTR exon	10 (45.5%)	12	9 (1.17%)	760
5′ UTR intron	20 (76.9%)	6	4 (2.56%)	152
3′ UTR exon	16 (64.0%)	9	9 (0.45%)	2000
3′ UTR intron	3 (100%)	0	2 (3.84%)	50
**Total**	**66 (53.7%)**	**57**	**24 (0.71%)**	**3340**

### Burden testing does not detect a significant enrichment of variants with any collective region or variant annotation

Given that we were able to identify disease-causing promoter and UTR variants using our region-based filtering pipeline, we sought to further use this approach to quantify the enrichment of potentially damaging promoter and UTR variants. Ideally such an approach would also utilise de novo variants that have a high prior probability of being pathogenic; however, there are only a small number of trios within GEL with an unaffected child, and mutational models to directly assess enrichment of de novo variants (by comparing observed to expected numbers) have not been well calibrated for non-coding regions, specifically struggling with the 5′ end of genes [75]. We instead used the full aggregated set of variants in GEL (which will include both inherited and de novo variants).

**Fig 3:**
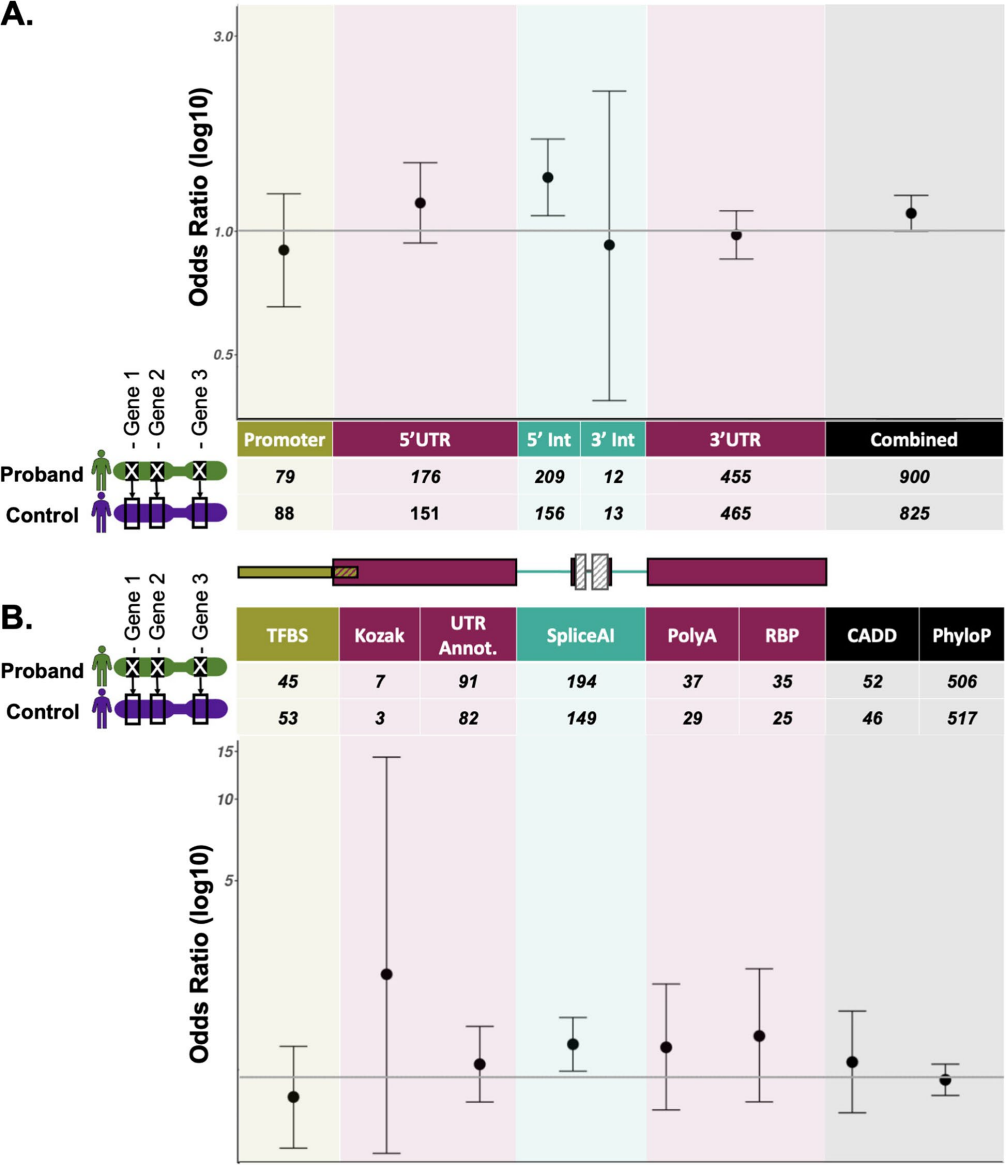
Burden testing results. Counts of variants and odd ratios (log10) testing for an enrichment of variants in cases compared to matched control participants, collectively by **A** region annotation and **B** variant annotation. Annotation groups with fewer than 10 participants are omitted. Error bars represent 95% confidence intervals. Variants in 5′UTRs (one-sided Fishers, *P*=0.016) and variants with SpliceAI ≥0.5 (one-sided Fisher’s *P*=0.0039) are enriched in cases over matched controls, but neither remains significant after correcting for multiple testing (Bonferroni threshold adjusting for 16 tests =0.0031). Full results are in Additional file 2:Table S5. Error bars represent 95% confidence intervals from a two-sided Fisher’s test

We analysed a set of 7862 probands matched, with replacement, to 6371 unrelated unaffected individuals on sex and genetically inferred ancestry (see ‘ Methods’; 1295 matched controls were partnered with more than one proband). Each control individual was assigned the same dominant, green panelApp genes as had been assigned to the rare disease proband by GEL, allowing us to control for gene- and region-level differences in mutability (Figure 3).

For all individuals, we extracted variants from GEL’s aggregated variant dataset (AggV2) and filtered these using our region-specific criteria (Additional file 2:Fig S5). Given that we used a high sensitivity SpliceAI threshold to prioritise DNVs with a high prior of pathogenicity, this was raised to a stricter cutoff of 0.5 for this analysis. As we are not powered to analyse individual genes or gene-regions, we performed burden testing collectively across all prioritised variants with the same regional (e.g. 5′UTR) or variant-level (e.g. SpliceAI) annotations, across all participants and their assigned green genes. Whilst we observed a greater number of probands with prioritised variants compared with matched controls for the majority of regional and variant-level annotations, no annotations were significantly enriched for variants in cases after correcting for multiple testing (Figure 3; Additional file 2:Table S5). We also did not observe a significant enrichment when combining across all regions and variant annotations (*OR=*1.09, 95% *CI*=[0.981,1.210]), this difference was not significant according to a one-tailed Fisher’s exact test (*P=*0.051).

We hypothesised that the lack of detectable burden in cases over controls is likely due to low variant numbers and limited statistical power. Indeed, at the current dataset size of 7862 cases and controls we estimate that we have 14.3% power to identify a true association with OR=1.09 (the estimate for our combined test across all region and variant annotations) at our study-wide *P*-value threshold of <0.0031. Assuming a constant ratio between case and control variants, and hence OR, we would need an estimated 7928 cases and controls for significance at *P*<0.05 in the combined test, and 21,528 for significance at *P*<0.0031 (Additional file 2:Fig S6).

We additionally applied our approach to de novo variants identified in 4199 trios from the SFARI SSC dataset (2317 individuals with autism and 1882 sibling controls). We identified 1678 variants (920 in cases and 766 in controls) in promoter and UTR regions of 664 NDD associated genes (Fu et al). One thousand five hundred and three of these variants appeared at a frequency of ≤0.00005 in gnomAD (833 in cases and 677 in controls). After annotation, we prioritised only six variants, four in cases and two in controls (Additional file 2:Table S9; Fisher’s *P*=0.698).

## Discussion

Here, we have described a framework for the identification and annotation of potentially disease-causing UTR and promoter variants in individuals with rare disease. We show the utility of the approach through identification of ten likely diagnoses in the GEL 100,000 genomes project rare disease cohort. These comprised three new confirmed diagnoses (*SLC2A1* and 2x *SETD5*) and three new likely diagnoses (*GNAS, NIPBL, ZBTB18*) alongside four previously confirmed diagnostic variants (2x *PAX6*, *RPL26*, and *MEF2C*). Whilst our results illustrate that expanding diagnostic screening into promoter regions and UTRs of known disease genes can identify new diagnoses, they suggest that the associated increase in diagnostic yield is likely to be very modest.

In our analysis, we concentrated on variants within or directly adjacent to UTR exons and proximal promoter sequences for three key reasons: (1) the functional link between these regions and the impacted gene is relatively clear; (2) the importance of these regions in gene regulation means that variants within them can have a large impact, even causing complete loss-of-function; and (3) known functional elements within these regions enable us to predict some variant effects. Many of these criteria do not apply to more distal non-coding elements, such as enhancers, which also suffer from redundancy, meaning small variants in any one enhancer may often be unlikely to have a large impact on gene expression and hence disease [76], although there are exceptions [77]. Recent work has, however, shown that variants impacting tissue-specific silencer elements may be a frequent cause of some disorders, indicating that these specific elements may have lower levels of redundancy [78, 79]. In addition, studies have demonstrated the importance of larger structural variants that disrupt cis-regulatory elements or chromatin conformation [80], deep intronic variants that impact splicing [81], and variants in non-coding RNAs in disease [82]. More research is needed to clarify the contribution of variants across diverse non-coding elements to rare monogenic disorders.

A key barrier to routine identification of non-coding variants in clinical settings is the potential dramatic increase in interpretation burden. Here, we employed strict filters based on known regulatory mechanisms, aiming for high specificity. Consequently, a very large proportion of the shortlisted variants (~82%) were flagged as good diagnostic candidates following clinical review. We also demonstrated this high specificity using ClinVar, where only 24 out of 3364 (0.71%) benign variants were prioritised by our approach. Conversely, our approach had reasonable sensitivity, correctly prioritising 66/123 (53.7%) ClinVar pathogenic variants, although we note that there could be some circularity in this analysis as our annotations (for example cross species conservation) likely contributed to the initial identification of these variants. Together, this illustrates the validity of our method as a highly specific route to finding new diagnoses without dramatically increasing the number of variants that need to be manually reviewed.

Here, we focus on de novo variants given their high prior probability of being pathogenic. Currently, de novo inheritance pattern, clinical fit, and functional validation are essential to identifying and classifying non-coding variants as (likely) pathogenic. Hence it is much harder to identify disease-causing non-coding variants in more heterogeneous conditions and/or disorders where de novo variants are not a frequent disease mechanism. However, the same annotation approach can be applied to inherited variants [83]. Additionally, although we restricted our burden testing approach to only individuals of European or South Asian genetic ancestry, this was only to facilitate participant matching. There is no reason that our approach would not work equally well across more heterogeneous populations.

Despite our strict filtering approach, the relatively modest number of new diagnoses given the size of the GEL cohort suggests that the proportion of currently undiagnosed individuals that will likely be diagnosed through regular assessment of proximal promoter and UTR regions will also be modest. This is in-line with the conclusions of our recent work looking for non-coding variants in recessive disease genes [83]. Nevertheless, our diagnostic yield is likely an underestimate. First, we limited our analyses to only genes within a diagnostic panel applied to each individual and, within this, we focused on genes with a clear dominant disease mechanism. Gene agnostic approaches may have greater sensitivity for new diagnoses and allow the identification of candidate novel disease genes. Our study was also limited to MANE transcripts and may miss important variants impacting alternate transcripts. Our strict filtering approach was necessitated by our limited understanding of the ‘regulatory genetic code’, and the paucity of tools to accurately determine non-coding variant deleteriousness, and likely excluded some important variants. For example, our analysis of ClinVar variants demonstrated a particularly low sensitivity of our approach to identify (likely) pathogenic promoter variants. Future work should focus on accessible tools that improve our ability to predict the pathogenicity of promoter variants at scale. Finally, we only removed individuals flagged as solved’ in the GEL ‘exit questionnaire’ as having an existing coding diagnosis. Many more individuals may be annotated as ‘partially solved’, or subsequently had likely diagnostic variants returned that were not reflected in the exit questionnaire at the time of analysis, due to ongoing analyses of the cohort. It is possible that the inclusion of these individuals in our analysis may result in the underestimation of diagnostic yield.

Amongst our novel diagnoses was a 5′UTR uAUG-creating variant in *SLC2A1*, which causes GLUT1 deficiency syndrome, which is treatable through diet. Hence, our diagnosis changed the clinical management of this patient. The exact same variant was found in a patient with a similar phenotype in 2017 [3], the same year the patient was recruited to GEL, but whilst the variant was deposited in the more specialist Leiden Open Variation Database [84] (I.D: SLC2A1_000036), it did not appear in the more widely used ClinVar database until 2022 (ID:1491299). This highlights the necessity of data sharing through variant databases and the use of these datasets for re-analysis to reduce the lengthy diagnostic odysseys so often faced by individuals with rare disease.

Whilst we expected the excess burden of promoter and UTR variants in cases to be relatively low, our approach was imperfect. In particular, analysing all variants identified in each individual (i.e. inherited as well as de novo) across large gene panels likely added a lot of noise. A better approach to assess this enrichment would be using only de novo variants; however, the number of trios within GEL where the child is unaffected is very small, and we and others have struggled to correctly optimise mutational models for application at the 5′ end of genes [75]. Multiple additional factors also likely contribute to our observed lack of signal. Firstly, we used unaffected parents of rare disease probands as a control and these individuals may be more likely to have damaging variants (for example variants with reduced penetrance, or variants that modify coding variant penetrance). Secondly, the sizes of gene panels varied substantially between participants, with some containing vast numbers of genes (Additional file 2:Fig S4). These larger panels likely contribute an overrepresentation of variants that are unlikely to be causal. Thirdly, whilst we devised a careful approach to annotation of both regulatory regions and potentially deleterious variants within them, we were limited by current knowledge of variant effects and available data for region definition. For example, we used a broad approach to annotate promoter regions and focused only on MANE transcripts; however, promoter and TSS usage are known to vary across and within tissues. It is possible that these factors may result in an underestimation of the burden of rare, causal variants in these regions. Future work should expand region definition across transcripts, use tissue-specific datasets, and include well-documented regulatory elements curated from the literature.

## Conclusions

Our understanding of the mechanisms that underlie variation in the non-coding genome is far from complete. Despite this, routine interrogation of these regions with existing knowledge and tools can return valuable genetic diagnoses to patients. Identifying more disease-causing variants in non-coding regions and understanding how they lead to disease will, in turn, increase our understanding of regulatory biology, and enable us to create better tools to identify and annotate these variants. Here, focusing specifically on proximal promoters, UTRs, and UTR introns, we developed a flexible approach for variant annotation and filtering which can be extended and adapted to incorporate new functional variant classes as our understanding of non-coding genome biology increases. Our framework provides a foundation for the systematic analysis of variants in these regions, which can be readily applied to cohorts, and in clinical settings, globally.

## Supplementary Information

Supplementary Material 1.

## Data Availability

The datasets supporting the conclusions of this article are available in the ‘near-coding annotation’ github repository [85]. This repository includes data and scripts relating to MANE transcripts of ‘green’ PanelApp genes with a dominant mode of inheritance (GRCh38), including; .tsv, .bed and .bb files containing the coordinates of UTR and promoter regions; The coordinates of annotation features not already available via the Ensembl VEP in .tsv format; And all scripts used to annotate near coding variants that fall within regulatory elements (https://github.com/Computational-Rare-Disease-Genomics-WHG/Near_coding_annotation). In addition, a UCSC genome browser public session entitled ‘CRDG Near coding regions’ Martin-Geary et al ‘24’ has been made available. This session contains a custom track showing near coding regions, including UTR exons, introns, and promoter regions in PanelApp green genes, and can be accessed via the UCSC Public Sessions portal: https://genome.ucsc.edu/cgi-bin/hgPublicSessions. This research was made possible through access to data in the National Genomic Research Library, which is managed by Genomics England Limited (a wholly owned company of the Department of Health and Social Care). The National Genomic Research Library holds data provided by patients and collected by the NHS as part of their care and data collected as part of their participation in research. All data from the Genomics England 100k Genomes Project v15 (26.05.2022), including Genetic, phenotypic and RNA-seq data, was accessed via the Genomics England Trusted Research Environment (TRE) and is available to registered users of the National Genomics Research Library through the TRE platform. Detailed methylation data used in this analysis cannot be shared due to EpiSign-related proprietary issues and legal constraints on the redistribution of NHS patient data of this kind. If required, part of these data may be made available upon request subject to gaining the necessary approvals.

## Abbreviations

AB: Allelic balance
AC: Allele count
AD: Autosomal dominant
AF: Allele frequency
AR: Autosomal recessive
cCRE: Candidate Cis Regulatory Element
CDS: Coding sequence
DHS: DNase I hypersensitive
DNV: De novo variant
dORF: Downstream Open Reading Frame
DP: Read depth
GEL: Genomics England 100,000 Genomes Project
GQ: Genotype Quality
HPO: Human Phenotype Ontology
IRES: Internal ribosome entry site
LoF: Loss of function
miRNA: MicroRNA
MOI: Mode of Inheritance
NS: Not specified
oORF: Overlapping Open Reading Frame
RBP: RNA-binding protein
TFBS: Transcription factor binding site
TSS: Transcription start site
uAUG: Upstream start codon
uORF: Upstream Open Reading Frame
UTR: Untranslated region
VEP: Variant effect predictor
