# Supplementary material for: Systematic identification of disease-causing promoter and untranslated region variants in 8040 undiagnosed individuals with rare disease
Source: Genome Med. 2025 Apr 14;17:40. doi: 10.1186/s13073-025-01464-2 (PMC11998461; doi:10.1186/s13073-025-01464-2)
Supplement: Supplementary file 1 — Supplementary Material 1. [file 13073_2025_1464_MOESM1_ESM.docx]

**Supplementary section**

**Supplementary Tables**

**Table S1**: Total, and mean size of near-coding region annotations by type.

| **Region** | **Total (bp)** | **Mean per gene (bp)** |
| --- | --- | --- |
| Promoter | 543,422 | 347.9 |
| 5’UTR | 372,417 | 239.2 |
| 5’UTR intron | 15,795,406 | 10080.0 |
| 3’UTR | 3,437,362 | 2194.7 |
| 3’UTR intron | 263,304 | 168.0 |
| All Regions | 20,409,032 | 13029.8 |

**Table S2**: Genomic coordinates of analysed near-coding regions across green dominant PanelApp genes.

Hosted on GitHub due to size: <https://github.com/Computational-Rare-Disease-Genomics-WHG/Near_coding_annotation>

**Table S3**: Region level annotations and resources contributing to each annotation. Where annotations were gained using 3rd party software this has been included in brackets.

| **Region** | **Source** | **Annotation** | **Type** | **Reference** |
| --- | --- | --- | --- | --- |
| All Regions | CADD v1.6 (VEP v99.1) | CADD | Variant | Rentzsh *et al* (2021)(26) |
| All Regions | PhyloP 100 way vertebrate (VEP v99.1) | PhyloP | Variant | Pollard *et al* (2010)(27) |
| Promoter | ENCODEv3 acc. ENCSR695MET+ FABIAN | TFBS | Variant | Vierstra *et al* (2020)(41)  Steinhaus *et al* (2022)(42) |
| 5’UTR | In-house (scripts on GitHub) | Kozak | Variant | <https://github.com/Computational-Rare-Disease-Genomics-WHG/Near_coding_annotation> |
| 5’UTR | IRESbase | IRES | Variant | Zhao *et al* (2020)(31) |
| 5’UTR | UTRannotator (VEP v99.1) | uORF | Variant | Zhang *et al* (2021)(29) |
| Introns | SpliceAI v1.3 (VEP v99.1) | Splicing | Variant | Jaganathan *et al* (2019)(NA) |
| 3’UTR | In-house (available via GitHub) | PolyA motif | Variant | <https://github.com/Computational-Rare-Disease-Genomics-WHG/Near_coding_annotation> |
| 3’UTR | Findlay *et al* | RBPs | Variant | Findlay *et al* (2022)(38) |
| 3’UTR | Chothani *et al* | dORF | Variant | Chothani *et al* (2022)(36) |
| 3’UTR | Literature | miRNA | Variant | Plotnikova *et al* (2019(32); Nowakowski *et al* (2018)(33);  Spengler *et al* (2016)(34);  Bodreau *et al* (2014)(35) |
| UTR exons & introns | MANE v1.0 | Region definition | Region | Morales *et al* (2022)(17) |
| Promoter | ENCODEv3 acc. ENCFF379UDA | Region definition | Region | Moore *et al* (2020)(18) |

**Table S4**: Total and mean variants across promoters and full MANE transcript length, in case and control participants in matched pairs, by genetically inferred ancestry group, and sex (AF 0.0001).

| **Sex** | **Genetically inferred ancestry** | **Case participants** | **Control participants** | **Mean Case variants** | **Mean Control variants** |
| --- | --- | --- | --- | --- | --- |
| Female | European | 7,827 | 5,198 | 430.38 | 428.04 |
| Male | European | 8,096 | 4,881 | 430.47 | 427.91 |
| Female | South Asian | 729 | 538 | 619.10 | 629.93 |
| Male | South Asian | 989 | 610 | 629.94 | 631.29 |

**Table S5**: Burden testing of prioritised variants in case and control participants by region and variant annotation. The proportion of total participants (N=7,862) are shown in the control, and case participant columns in round brackets. *P*-values were calculated using a one-sided Fisher’s test, assessing for an increase in prioritised variants in cases over controls. A Bonferroni adjusted P-value threshold of 0.0031 correcting for 16 tests was used to assess statistical significance. 95% confidence intervals from a two-sided Fisher’s test are shown in square brackets alongside the odds ratios.

| **Region** | **Control Participants** | **Case Participants** | **Fisher’s *P*-value** | **Odds Ratio** |
| --- | --- | --- | --- | --- |
|  |  |  |  |  |
| ALL | 770 (9.79%) | 833 (10.59%) | 0.051 | 1.091 [0.982,1.212] |
| Promoter | 107 (1.36%) | 105 (1.34%) | 0.582 | 0.981 [0.741,1.299] |
| 5’UTR | 164 (2.09%) | 182 (2.31%) | 0.177 | 1.112 [0.894,1.386] |
| 5’UTR intron | 103 (1.31%) | 137 (1.74%) | 0.016 | 1.336 [1.025,1.746] |
| 3’UTR | 465 (5.91%) | 455 (5.78%) | 0.645 | 0.977 [0.853,1.118] |
| 3’UTR intron | 3 (0.04%) | 11 (0.14%) | 0.029 | 3.670 [0.969,20.494] |
| **Annotation** |  |  |  |  |
| CADD | 38 (0.48%) | 57 (0.73%) | 0.031 | 1.504 [0.979,2.333] |
| PhyloP | 462 (5.88%) | 473 (6.02%) | 0.367 | 1.025 [0.896,1.173] |
| TFBS | 53 (0.67%) | 51 (0.65%) | 0.615 | 0.962 [0.641,1.442] |
| KOZAK | 3 (0.04%) | 7 (0.09%) | 0.171 | 2.335 [0.533,13.995] |
| IRES | 0 (0.00%) | 1 (0.01%) | 0.499 |  |
| UTR Annotator | 82 (1.04%) | 94 (1.20%) | 0.202 | 1.148 [0.843,1.566] |
| SpliceAI | 149 (1.90%) | 199 (2.53%) | 3.9x10^-3^ | 1.344 [1.079,1.678] |
| PolyA | 29 (0.36%) | 37 (0.47%) | 0.194 | 1.277 [0.763,2.155] |
| RBP | 25 (0.32%) | 35 (0.45%) | 0.122 | 1.402 [0.815,2.446] |
| dORF | 7 (0.09%) | 2 (0.03%) | 0.980 |  |

**Table S6**: Classifications for the six newly identified DNVs according to the ACMG/AMP guidelines and adaptations for variants in non-coding regions[(8,61)](https://paperpile.com/c/mlVjvG/8xFZ+7Xam). Thresholds for PP3 were taken from Pejaver *et al*. 2022 (doi: 10.1016/j.ajhg.2022.10.013) for PhyloP and CADD, and Walker *et al*. 2023 (doi: 10.1101/2023.02.24.23286431) for SpliceAI. *Functional data from Willemsen *et al*. 2017 (doi: 10.1038/ejhg.2017.45).

| **Variant** | **Gene** | **Applied rule codes** | **Classification** |
| --- | --- | --- | --- |
| chr1:g.244051270 C>T | *ZBTB18* | PS2 (de novo); PP3_supporting (PhyloP score); PM2_supporting (absent from gnomADv3.1.2) | Likely Pathogenic |
| chr1:g.42958758 C>T | *SLC2A1* | PS2 (de novo); PP3_supporting (UTRannotator prediction); PM2_supporting (absent from gnomADv3.1.2); PS3_supporting (published functional studies*) | Likely Pathogenic |
| chr3:g.9397978  G>A | *SETD5* | PS2 (de novo); PP3_moderate (SpliceAI score); PM2_supporting (absent from gnomADv3.1.2); PP4_supporting (methylation studies confirm *SETD5*); PS1_supporting (same nucleotide) | Likely Pathogenic |
| chr3:g.9397974 CAAGGT>C | *SETD5* | PS2 (de novo); PP3_moderate (SpliceAI score); PM2_supporting (absent from gnomADv3.1.2); PP4_supporting (methylation studies confirm *SETD5*); PS1_supporting (same nucleotide) | Likely Pathogenic |
| chr5:g.36953601 T>A | *NIBPL* | PS2 (de novo); PP3_moderate (SpliceAI score); PM2_supporting (absent from gnomADv3.1.2) | Likely Pathogenic |
| chr20:g.58909654 A>G | *GNAS* | PS2 (de novo); PM2_supporting (absent from gnomADv3.1.2); PS3_supporting (evidence of aberant splicing and significant reduction in expression compared to 499 controls shown in RNA-seq) | Likely Pathogenic |

**Table S7:** ClinVar promoter and UTR pathogenic variants correctly annotated by our pipeline.

| **Variant** | **Annotation/ Details** | **Location** | **Transcript** | **Gene** | **gnomAD < 5×10^-5^** |
| --- | --- | --- | --- | --- | --- |
| 1:93993244_T/C | SpliceAI:0.98\|CADD:33 | 3' UTR intron | ENST00000370225 | ABCA4 | Y |
| 1:75724819_T/C | CADD:34 | promoter | ENST00000370841 | ACADM | Y |
| X:15854687_C/T | SpliceAI:0.98\|CADD:33 | 5' UTR intron | ENST00000672987 | AP1S2 | Y |
| 6:131573340_G/A | CADD:33 | promoter | ENST00000368087 | ARG1 | Y |
| 15:72686252_G/T | CADD:34 | promoter | ENST00000268057 | BBS4 | Y |
| 13:32316419_CAG/C | SpliceAI:0.97 | 5' UTR intron | ENST00000380152 | BRCA2 | Y |
| 13:32316420_A/G | SpliceAI:0.97\|CADD:34 | 5' UTR intron | ENST00000380152 | BRCA2 | Y |
| 13:32316421_G/C | SpliceAI:0.97\|CADD:34 | 5' UTR intron | ENST00000380152 | BRCA2 | Y |
| 17:4899385_C/G | CADD:27.3 | promoter | ENST00000381365 | C17orf107 | Y |
| 17:4899386_T/A | CADD:33 | promoter | ENST00000381365 | C17orf107 | Y |
| 17:4902328_T/C | PHYLOP:8.01\|CADD:34 | 3' UTR exon | ENST00000381365 | C17orf107 | Y |
| 11:59845374_C/T | CADD:32 | promoter | ENST00000257248 | CBLIF | Y |
| 1:100249769_C/A | CADD:35 | promoter | ENST00000370132 | DBT | Y |
| 18:31498297_G/A | CADD:34 | promoter | ENST00000261590 | DSG2 | Y |
| 7:6023301_A/G | PHYLOP:9.32\|CADD:34 | 3' UTR exon | ENST00000199389 | EIF2AK1 | Y |
| 19:48965340_G/C | PHYLOP:9.53 | 5' UTR exon\|promoter | ENST00000331825 | FTL | Y |
| 19:48965340_G/T | PHYLOP:9.53 | 5' UTR exon\|promoter | ENST00000331825 | FTL | Y |
| 19:48965341_C/T | PHYLOP:7.54 | 5' UTR exon\|promoter | ENST00000331825 | FTL | Y |
| 19:48965358_C/A | PHYLOP:7.54 | 5' UTR exon\|promoter | ENST00000331825 | FTL | Y |
| 17:80104553_A/G | SpliceAI:0.98 | 5' UTR intron | ENST00000302262 | GAA | Y |
| 17:80104554_G/C | SpliceAI:0.98\|CADD:31 | 5' UTR intron | ENST00000302262 | GAA | Y |
| X:71223335_G/A | SpliceAI:0.36 | 5' UTR exon | ENST00000361726 | GJB1 | Y |
| 20:58891866_G/C | SpliceAI:0.99 | 3_prime_intron | ENST00000371075 | GNAS | Y |
| 20:58903792_G/A | SpliceAI:0.93\|PHYLOP:9.40\|CADD:34 | 3' UTR intron | ENST00000371075 | GNAS | Y |
| 20:34955722_C/T | SpliceAI:0.88 | 5' UTR intron\|promoter | ENST00000651619 | GSS | Y |
| 16:173008_G/A | CADD:35 | promoter | ENST00000251595 | HBA2 | Y |
| 16:173691_TAA/T | Predicted to disrupt PolyA site | 3' UTR exon | ENST00000251595 | HBA2 | Y |
| 16:173694_A/G | Predicted to disrupt PolyA site | 3' UTR exon | ENST00000251595 | HBA2 | Y |
| 15:72686252_G/T | CADD:34 | promoter | ENST00000311755 | HIGD2B | Y |
| 17:47253941_G/A | CADD:34 | promoter | ENST00000559488 | ITGB3 | Y |
| X:154097600_TCA/T | SpliceAI:1 | 5' UTR intron | ENST00000303391 | MECP2 | Y |
| X:154097602_A/C | SpliceAI:1\|CADD:34 | 5' UTR intron | ENST00000303391 | MECP2 | Y |
| X:154097603_C/T | SpliceAI:0.95\|CADD:33 | 5' UTR intron | ENST00000303391 | MECP2 | Y |
| 1:45511415_C/A | PHYLOP:7.87\|CADD:35 | 3' UTR exon | ENST00000401061 | MMACHC | Y |
| 6:49459506_C/T | CADD:33 | 5' UTR intron | ENST00000274813 | MMUT | Y |
| 1:43337929_T/A | CADD:34 | promoter | ENST00000372470 | MPL | Y |
| 1:45334513_T/C | SpliceAI:0.99\|CADD:33 | 5' UTR intron | ENST00000456914 | MUTYH | Y |
| 1:45340218_C/T | CADD:25.3 | promoter | ENST00000456914 | MUTYH | Y |
| 1:45340219_C/T | CADD:36 | promoter | ENST00000456914 | MUTYH | Y |
| 1:45340220_C/T | CADD:33 | promoter | ENST00000456914 | MUTYH | Y |
| 11:47352622_C/T | CADD:33 | promoter | ENST00000545968 | MYBPC3 | Y |
| 15:48139176_G/A | PHYLOP:9.33\|CADD:33 | 3' UTR exon | ENST00000324324 | MYEF2 | Y |
| X:153929948_T/C | Predicted to disrupt PolyA site | 3' UTR exon | ENST00000464845 | NAA10 | Y |
| 20:3889410_G/T | CADD:35 | 5' UTR exon\|promoter | ENST00000610179 | PANK2 | Y |
| 11:31806913_TAA/T | 5_prime_UTR_stop_codon_loss_variant | 5' UTR exon | ENST00000640368 | PAX6 | Y |
| 11:31806926_CT/C | SpliceAI:0.65 | 5' UTR intron | ENST00000640368 | PAX6 | Y |
| 6:83191208_G/A | SpliceAI:0.27\|CADD:25.4 | 5_prime_intron | ENST00000513973 | PGM3 | Y |
| 7:6008996_C/A | CADD:34 | promoter | ENST00000265849 | PMS2 | Y |
| 12:56042170_G/T | SpliceAI:0.99\|CADD:34 | 5' UTR intron | ENST00000646449 | RPS26 | Y |
| 12:56042171_T/G | SpliceAI:0.99\|CADD:33 | 5' UTR intron | ENST00000646449 | RPS26 | Y |
| 17:28402322_C/T | CADD:26.6 | 3' UTR exon | ENST00000585482 | SARM1 | Y |
| 11:112086960_G/A | CADD:35 | promoter | ENST00000375549 | SDHD | Y |
| 17:50166078_G/C | CADD:29.4 | promoter | ENST00000262018 | SGCA | Y |
| 5:149960981_T/C | SpliceAI:0.9\|CADD:33 | 5' UTR intron | ENST00000286298 | SLC26A2 | Y |
| 1:42958633_C/T | SpliceAI:0.99\|CADD:34 | 5' UTR intron | ENST00000426263 | SLC2A1 | Y |
| 15:64156910_C/A | PHYLOP:7.43\|CADD:35 | 3' UTR exon | ENST00000325881 | SNX22 | Y |
| 11:112086960_G/A | CADD:35 | promoter | ENST00000504148 | TIMM8B | Y |
| 11:61392667_T/C | CADD:34 | promoter | ENST00000515837 | TMEM216 | Y |
| 17:16972014_C/T | CADD:32 | promoter | ENST00000261652 | TNFRSF13B | Y |
| 1:45340218_C/T | SpliceAI:0.88\|CADD:25.3 | 5' UTR exon\|promoter | ENST00000372090 | TOE1 | Y |
| 1:45340219_C/T | SpliceAI:0.82\|CADD:36 | 5' UTR exon\|promoter | ENST00000372090 | TOE1 | Y |
| 1:45340220_C/T | SpliceAI:0.22\|CADD:33 | 5' UTR exon\|promoter | ENST00000372090 | TOE1 | Y |
| 11:6619382_A/T | SpliceAI:0.98\|CADD:34 | 5' UTR intron\|promoter | ENST00000299427 | TPP1 | Y |
| 12:49188973_TC/T | SpliceAI:0.41 | 5' UTR intron | ENST00000301071 | TUBA1A | Y |
| 16:89738710_T/C | CADD:31 | 3' UTR exon | ENST00000443381 | ZNF276 | Y |
| 16:89738710_T/G | CADD:31 | 3' UTR exon | ENST00000443381 | ZNF276 | Y |
| 16:89738881_C/T | CADD:27 | 3' UTR exon | ENST00000443381 | ZNF276 | Y |
| 16:89738976_T/C | CADD:33 | 3' UTR exon | ENST00000443381 | ZNF276 | Y |
| 16:89739476_A/G | CADD:28.4 | 3' UTR exon | ENST00000443381 | ZNF276 | Y |
| 16:89739554_C/A | CADD:29.3 | 3' UTR exon | ENST00000443381 | ZNF276 | Y |
| 16:89739992_A/G | CADD:25.9 | 3' UTR exon | ENST00000443381 | ZNF276 | Y |
| 1:93993244_T/C | SpliceAI:0.98\|CADD:33 | 3' UTR intron | ENST00000370225 | ABCA4 | Y |

**Table S8:** ClinVar promoter and UTR variants incorrectly annotated by our pipeline. All missed pathogenic variants, and erroneously prioritised benign variants are included.

| **Variant** | **ClinVar Path.** | **Reason for misannotation** | **Location** | **Transcript** | **Gene** | **gnomAD < 5×10^-5^** |
| --- | --- | --- | --- | --- | --- | --- |
| 1:99851033_A/G | Benign | Prioritised by SpliceAI (0.24) | 5' UTR exon | ENST00000361915 | AGL | Y |
| 1:108930191_T/C | Benign | Prioritised by SpliceAI (0.24) | 3' UTR exon | ENST00000369969 | CLCC1 | Y |
| 16:28477361_T/G | Benign | Prioritised by SpliceAI (0.26) | 3' UTR exon | ENST00000636147 | CLN3 | Y |
| 19:55166518_C/A | Benign | Prioritised by SpliceAI (0.69) | 5' UTR intron | ENST00000524407 | DNAAF3 | Y |
| 21:37420393_A/G | Benign | Prioritised by SpliceAI (0.24) | 5' UTR intron | ENST00000647188 | DYRK1A | Y |
| X:48521910_G/C | Benign | Prioritised by SpliceAI (0.47) | 5' UTR intron\|promoter | ENST00000495186 | EBP | Y |
| 1:147759265_A/G | Benign | Prioritised by SpliceAI (0.23) | 5' UTR exon | ENST00000579774 | GJA5 | Y |
| 1:108930191_T/C | Benign | Prioritised by SpliceAI (0.24) | 3' UTR exon | ENST00000264126 | GPSM2 | Y |
| 1:119748835_T/G | Benign | Prioritised by SpliceAI (0.43) | 3' UTR exon | ENST00000369406 | HMGCS2 | Y |
| X:28789324_C/T | Benign | UTR Annotator predicted upstream start gain | 5' UTR exon | ENST00000378993 | IL1RAPL1 | Y |
| 1:1615612_C/A | Benign | Prioritised by CADD (25.3) | 5' UTR exon | ENST00000355826 | MIB2 | Y |
| 20:10405209_T/C | Benign | Predicted to disrupt PolyA site | 3' UTR exon | ENST00000347364 | MKKS | Y |
| 15:34341923_C/G | Benign | Prioritised by SpliceAI (0.22) | 3' UTR exon | ENST00000328848 | NOP10 | Y |
| 12:7210243_G/C | Benign | Prioritised by SpliceAI (0.7) and CADD (25.9) | 3' UTR exon | ENST00000675855 | PEX5 | Y |
| 12:7210364_G/A | Benign | Prioritised by SpliceAI (0.22) | 3' UTR exon | ENST00000675855 | PEX5 | Y |
| X:15335513_T/C | Benign | UTR Annotator predicted upstream start loss | 5' UTR exon\|promoter | ENST00000333590 | PIGA | Y |
| 6:43517095_G/A | Benign | UTR Annotator predicted upstream start gain | 5' UTR exon\|promoter | ENST00000642195 | POLR1C | Y |
| 17:58692637_G/A | Benign | Prioritised by SpliceAI (0.63) | 5' UTR exon\|promoter | ENST00000337432 | RAD51C | Y |
| 20:19889577_G/A | Benign | UTR Annotator predicted upstream start gain | 5' UTR exon | ENST00000255006 | RIN2 | Y |
| 12:20815731_GTTTA/G | Benign | Prioritised by SpliceAI (0.29) | 5' UTR exon | ENST00000381545 | SLCO1B3 | Y |
| 11:61398259_C/CA | Benign | Prioritised by SpliceAI (0.93) | 3' UTR intron | ENST00000515837 | TMEM216 | Y |
| 11:61398269_G/C | Benign | Prioritised by SpliceAI (0.79) | 3' UTR intron | ENST00000515837 | TMEM216 | Y |
| 21:42395475_C/G | Benign | Prioritised by SpliceAI (0.26) | 5' UTR intron | ENST00000644384 | TMPRSS3 | Y |
| X:123907193_A/G | Benign | Prioritised by SpliceAI (0.24) | 3' UTR exon | ENST00000371199 | XIAP | Y |
| 12:32747042_T/C | Benign | Predicted to disrupt PolyA site | 3' UTR exon | ENST00000324868 | YARS2 | Y |
| 12:6943843_C/T | Pathogenic | Promoter variant | promoter | ENST00000229281 | C12orf57 | Y |
| 12:6943848_AGGCTTTCT/A | Pathogenic | Promoter variant | promoter | ENST00000229281 | C12orf57 | Y |
| 19:29708415_G/A | Pathogenic | Kozak variant -2 position | 5' UTR exon | ENST00000323670 | C19orf12 | Y |
| X:86047479_C/T | Pathogenic | Promoter variant | promoter | ENST00000357749 | CHM | Y |
| 17:4903158_C/T | Pathogenic | Promoter variant, failed gnomAD allele frequency but recessive disease gene | promoter | ENST00000649488 | CHRNE | 7.25 × 10^-5^ |
| 19:48965347_C/T | Pathogenic | iron-responsive element (IRE) domain variant | 5' UTR exon\|promoter | ENST00000331825 | FTL | Y |
| 19:48965348_A/G | Pathogenic | iron-responsive element (IRE) domain variant | 5' UTR exon\|promoter | ENST00000331825 | FTL | Y |
| 17:80104542_T/G | Pathogenic | Missed splice variant SpliceAI score below 0.2 | 5' UTR intron | ENST00000302262 | GAA | Y |
| 17:80104552_C/A | Pathogenic | Missed splice variant SpliceAI score below 0.2 | 5' UTR intron | ENST00000302262 | GAA | Y |
| X:71223249_C/T | Pathogenic | IRES variant, does not pass CADD and PhyloP thresholds | 5' UTR exon\|promoter | ENST00000361726 | GJB1 | Y |
| 13:20192783_C/A | Pathogenic | Missed splice variant SpliceAI score below 0.2 & Failed gnomAD allele frequency but recessive disease gene | 5' UTR exon | ENST00000382848 | GJB2 | 1.24 × 10^-3^ |
| 13:20189605_T/G | Pathogenic | SpliceAI (0.91) CADD(33), failed gnomAD allele frequency but recessive disease gene | 5' UTR intron | ENST00000382848 | GJB2 | 9.15 × 10^-5^ |
| 13:20192782_C/T | Pathogenic | SpliceAI (1.0) \|CADD (25.5), failed gnomAD allele frequency but recessive disease gene | 5' UTR intron | ENST00000382848 | GJB2 | 2.27 x 10^-3^ |
| 1:228149860_A/G | Pathogenic | Promoter variant | promoter | ENST00000366714 | GJC2 | Y |
| 16:173692_A/G | Pathogenic | Polyadenylation variant, failed gnomAD allele frequency but recessive disease gene | 3' UTR exon | ENST00000251595 | HBA2 | 4.31 x 10^-4^ |
| 11:5227039_G/C | Pathogenic | MARE motif variant (TF) | 5' UTR exon\|promoter | ENST00000335295 | HBB | Y |
| 11:5227050_C/T | Pathogenic | UTRannotator predicted upstream start gain, failed gnomAD allele frequency but recessive disease gene | 5' UTR exon\|promoter | ENST00000335295 | HBB | 2.07 × 10^-4^ |
| 11:5227071_T/G | Pathogenic | Cap+1 variant (not considered), failed gnomAD allele frequency but recessive disease gene | 5' UTR exon\|promoter | ENST00000335295 | HBB | 4.14 × 10^-4^ |
| 11:5227099_T/C | Pathogenic | Promoter variant, failed gnomAD allele frequency but recessive disease gene | promoter | ENST00000335295 | HBB | 1.93 × 10^-4^ |
| 11:5227099_T/G | Pathogenic | Promoter variant | promoter | ENST00000335295 | HBB | Y |
| 11:5227100_T/C | Pathogenic | Promoter variant, failed gnomAD allele frequency but recessive disease gene | promoter | ENST00000335295 | HBB | 2.99 x 10^-3^ |
| 11:5227101_A/T | Pathogenic | Promoter variant | promoter | ENST00000335295 | HBB | Y |
| 11:5227102_T/C | Pathogenic | Promoter variant | promoter | ENST00000335295 | HBB | Y |
| 11:5227142_G/A | Pathogenic | Promoter variant | promoter | ENST00000335295 | HBB | Y |
| 11:5227157_G/C | Pathogenic | Promoter variant | promoter | ENST00000335295 | HBB | Y |
| 11:5227158_G/A | Pathogenic | Promoter variant, failed gnomAD allele frequency but recessive disease gene | promoter | ENST00000335295 | HBB | 2.08 × 10^-4^ |
| 11:5227158_G/C | Pathogenic | Promoter variant | promoter | ENST00000335295 | HBB | Y |
| 11:5227158_G/T | Pathogenic | Promoter variant, failed gnomAD allele frequency but recessive disease gene | promoter | ENST00000335295 | HBB | 1.21 × 10^-4^ |
| 11:5227159_G/A | Pathogenic | Promoter variant, failed gnomAD allele frequency but recessive disease gene | promoter | ENST00000335295 | HBB | 6.21 × 10^-4^ |
| 11:5227159_G/T | Pathogenic | Promoter variant | promoter | ENST00000335295 | HBB | Y |
| 11:5227161_G/A | Pathogenic | Promoter variant, failed gnomAD allele frequency but recessive disease gene | promoter | ENST00000335295 | HBB | 1.21 × 10^-4^ |
| 11:5227172_G/A | Pathogenic | Promoter variant | promoter | ENST00000335295 | HBB | Y |
| 12:120978486_A/C | Pathogenic | Promoter variant | promoter | ENST00000257555 | HNF1A | Y |
| 20:44355624_G/A | Pathogenic | Promoter variant | promoter | ENST00000316673 | HNF4A | Y |
| 14:58428236_A/C | Pathogenic | Missed splice variant SpliceAI score below 0.2 | 5' UTR exon | ENST00000652326 | KIAA0586 | Y |
| 19:11089400_C/A | Pathogenic | Promoter variant | promoter | ENST00000558518 | LDLR | Y |
| 19:11089413_C/T | Pathogenic | Promoter variant | promoter | ENST00000558518 | LDLR | Y |
| 19:11089414_C/G | Pathogenic | Promoter variant | promoter | ENST00000558518 | LDLR | Y |
| 11:77130654_T/A | Pathogenic | SplicaAI (0.98) CADD (33), failed gnomAD allele frequency but recessive disease gene | 5' UTR intron | ENST00000409709 | MYO7A | 2.07 × 10^-4^ |
| X:22248184_A/G | Pathogenic | Variant 3bp upstream of PolyA motif (Not considered) | 3' UTR exon | ENST00000379374 | PHEX | Y |
| 1:155301467_T/C | Pathogenic | Promoter variant | promoter | ENST00000342741 | PKLR | Y |
| 16:8797716_G/T | Pathogenic | Promoter variant | promoter | ENST00000268261 | PMM2 | Y |
| 11:47449174_T/C | Pathogenic | Promoter variant, failed gnomAD allele frequency but recessive disease gene | promoter | ENST00000298854 | RAPSN | 1.92 × 10^-4^ |
| 13:48303715_G/A | Pathogenic | Promoter variant | promoter | ENST00000267163 | RB1 | Y |
| 1:68449890_C/T | Pathogenic | Missed splice variant SpliceAI score below 0.2 in precomputed scores, but 0.87 when run directly using updated transcript definitions. | 5' UTR intron\|promoter | ENST00000262340 | RPE65 | Y |
| 6:158999809_T/TG | Pathogenic | Interpreted in ClinVar as a protein-coding premature termination codon variant, despite being in a UTR exon in all annotated transcripts in GRCh38. | 5' UTR exon | ENST00000367069 | RSPH3 | Y |
| 11:112086960_GT/AA | Pathogenic | Promoter variant | promoter | ENST00000375549 | SDHD | Y |
| X:630879_G/A | Pathogenic | Missed splice variant SpliceAI score below 0.2 | 5' UTR exon\|promoter | ENST00000686671 | SHOX | Y |
| 22:23834262_C/T | Pathogenic | Missed splice variant SpliceAI score below 0.2 in precomputed scores, but 0.71 when run directly using updated transcript definitions. | 3' UTR exon | ENST00000644036 | SMARCB1 | Y |
| 6:32853681_CC/AT | Pathogenic | Interpreted in ClinVar as a protein-coding premature termination codon variant, despite being in a UTR exon in all annotated transcripts in GRCh38. | 5' UTR exon\|promoter | ENST00000354258 | TAP1 | Y |
| 11:112086960_GT/AA | Pathogenic | Promoter variant | promoter | ENST00000504148 | TIMM8B | Y |
| 17:8173517_T/C | Pathogenic | RNA binding site variant (​​LSm) missed by our pipeline. | 3' UTR exon | ENST00000437139 | TMEM107 | Y |
| 17:8173581_C/G | Pathogenic | Not in SpliceAI precomputed scores. Max delta <0.2 when SpliceAI is run directly with updated transcript definitions | 3' UTR exon | ENST00000437139 | TMEM107 | Y |
| 16:8797716_G/T | Pathogenic | Promoter variant | promoter | ENST00000333050 | TMEM186 | Y |
| 13:38349764_TTCA/T | Pathogenic | Promoter variant | promoter | ENST00000239878 | UFM1 | Y |
| 16:89738880_A/AC | Pathogenic | Predicted to affect splicing of CDS exons in an alternative, overlapping gene (FANCA) | 3' UTR exon | ENST00000443381 | ZNF276 | Y |
| 16:89739459_GCCCTGTGGGTGGAGGTAC/G | Pathogenic | Not included in SpliceAI precomputed scores as is a large deletion.When SpliceAI is run directly with updated transcript definitions: 0.66 for FANCA, < 0.2 for ZNF276 (likely FANCA coding impact) | 3' UTR exon | ENST00000443381 | ZNF276 | Y |
| 16:89740803_C/G | Pathogenic | Splice variant only pathogenic in CDS intron in alternate gene (FANCA) | 3' UTR exon | ENST00000443381 | ZNF276 | Y |
| 16:89740803_C/T | Pathogenic | Splice variant only pathogenic in CDS intron in alternate gene (FANCA) | 3' UTR exon | ENST00000443381 | ZNF276 | Y |

**Table S9:** Prioritised variants from the Simons Foundation Autism Research Initiative (SFARI) Simplex Collection (SSC) cohort.

| **Variant** | **Region** | **Case/Control** | **Annotation** |
| --- | --- | --- | --- |
| Chr14:99272165 G>C | Promoter/5’UTR | Case | PhyloP (8.35) |
| Chr10:113167668 G>A | 3’UTR | Case | PhyloP (9.44) |
| Chr20: 10306498 G>A | 3’UTR | Case | PhyloP (9.16) |
| Chr1:61458082 G>A | 3’UTR | Control | PhyloP (8.15) |
| Chr12: 122143154 C>T | 3’UTR | Control | SpliceAI (0.54) |
| Chr4:82355303 C>T | 3’ Intron | Case | SpliceAI (0.99) |

**Supplementary Figures**


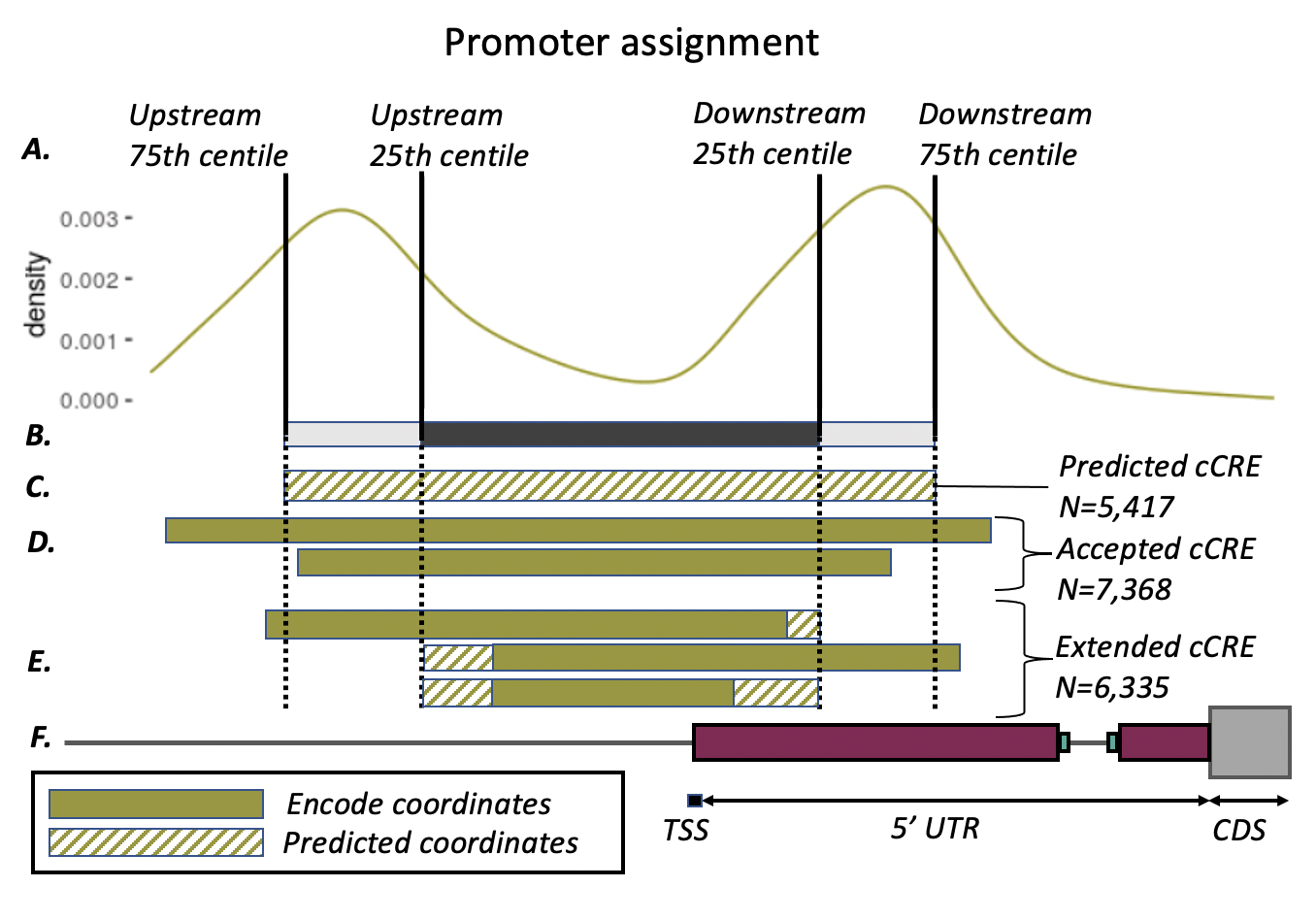
**Figure S1**: Graphical representation of promoter assignment with reference to the TSS (F). For all cCREs that overlapped a TSS the total distance the cCRE extends in each direction (upstream/5’ and downstream/3’) was calculated (A). The 25^th^ and 75^th^ percentiles of the distance in each direction was calculated to form the basis of a ‘minimal’ (B, dark grey) and ‘maximal’ (B, light grey) promoter. For genes without an overlapping cCRE a promoter was predicted using the ‘maximal’ promoter criteria (C, shaded line). For genes with an overlapping cCRE that extended beyond the 25^th^ centile in both directions (D) the cCRE was accepted without modification. For genes where the overlapping cCRE (E, solid line) fell short of the 25^th^ centile in either direction, this was extended to match the coordinates of the ‘minimal’ promoter (E, shaded line).

**
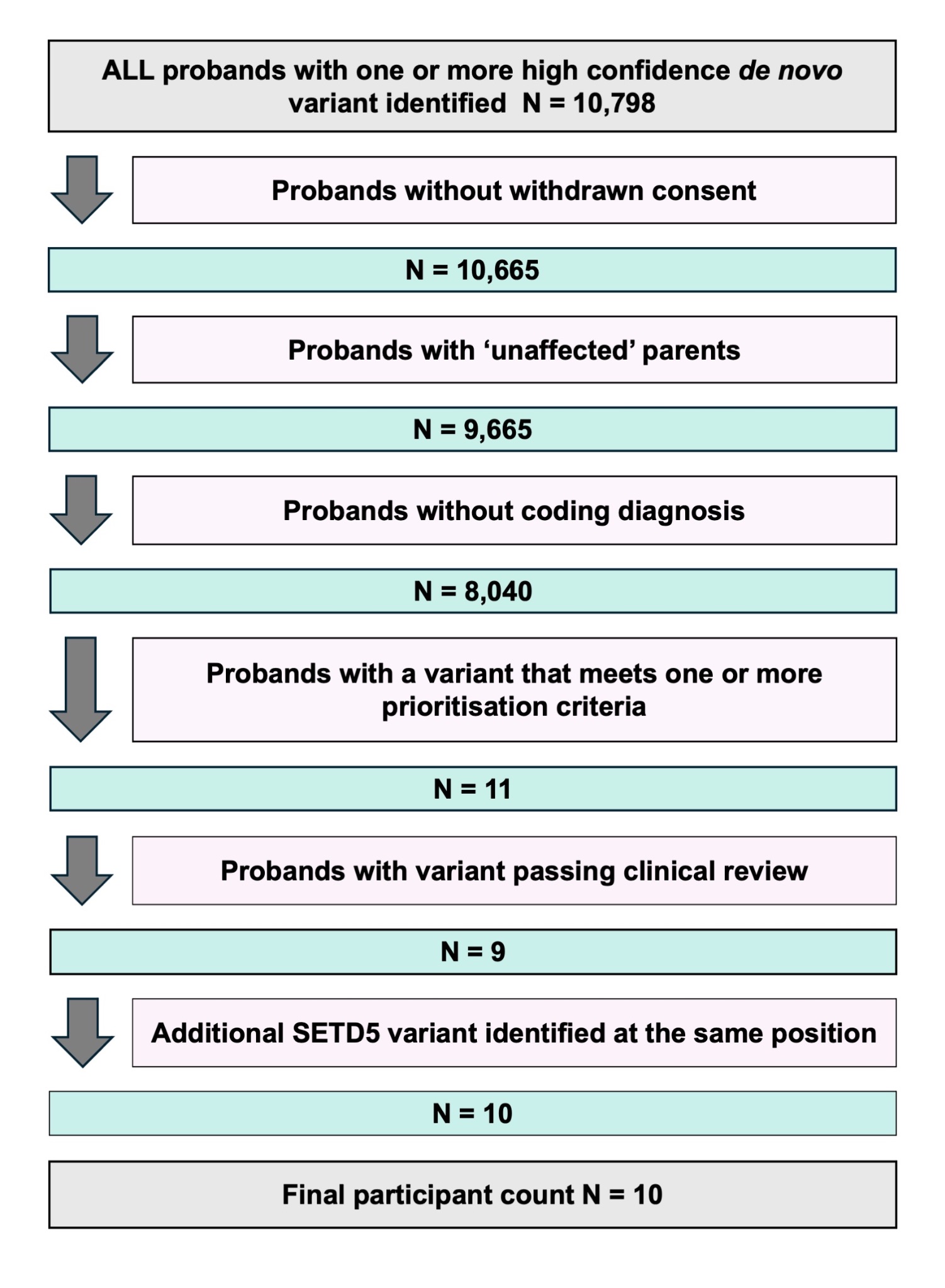
**

**Figure S2:** Flowchart showing *de novo* participant counts for all steps in our pipeline. Filtering steps are shown in pink boxes; Participant counts following each step are shown in green boxes. Initial and final participant counts are shown in grey boxes.


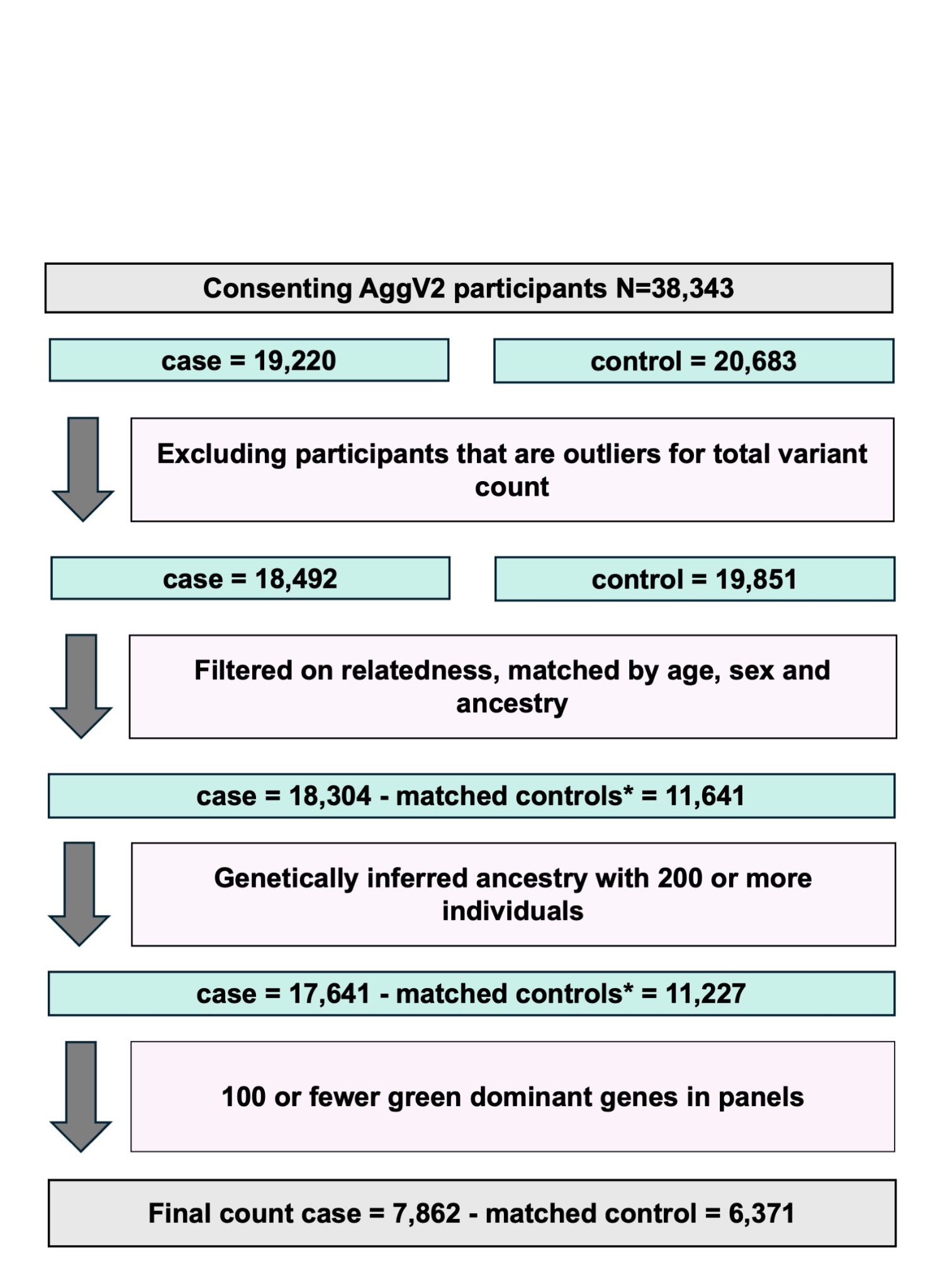


**Figure S3:** Flowchart showing Burden testing participant counts for all steps in our pipeline. Filtering steps are shown in pink boxes; Participant counts following each step are shown in green boxes. Initial and final participant counts are shown in grey boxes. Control participant counts are independent of Case counts, Matched control counts marked by * deplete in line with deletion of matched cases.

*
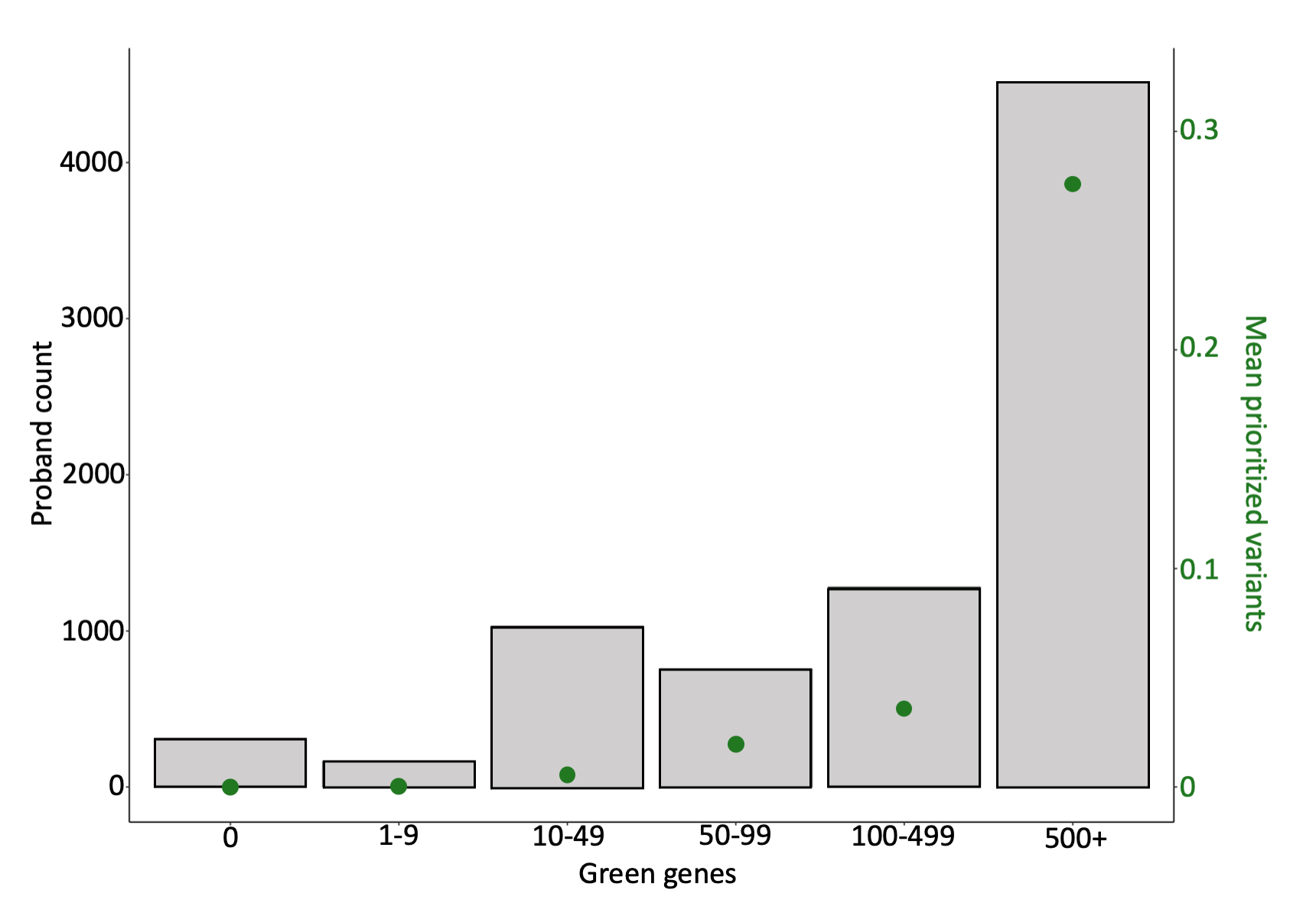
*

**Figure S4**: The number of probands with different numbers of assigned green-dominant genes (grey bars) and mean prioritised variants from the *de novo* variant dataset (green dots) in 8,040 probands.

*
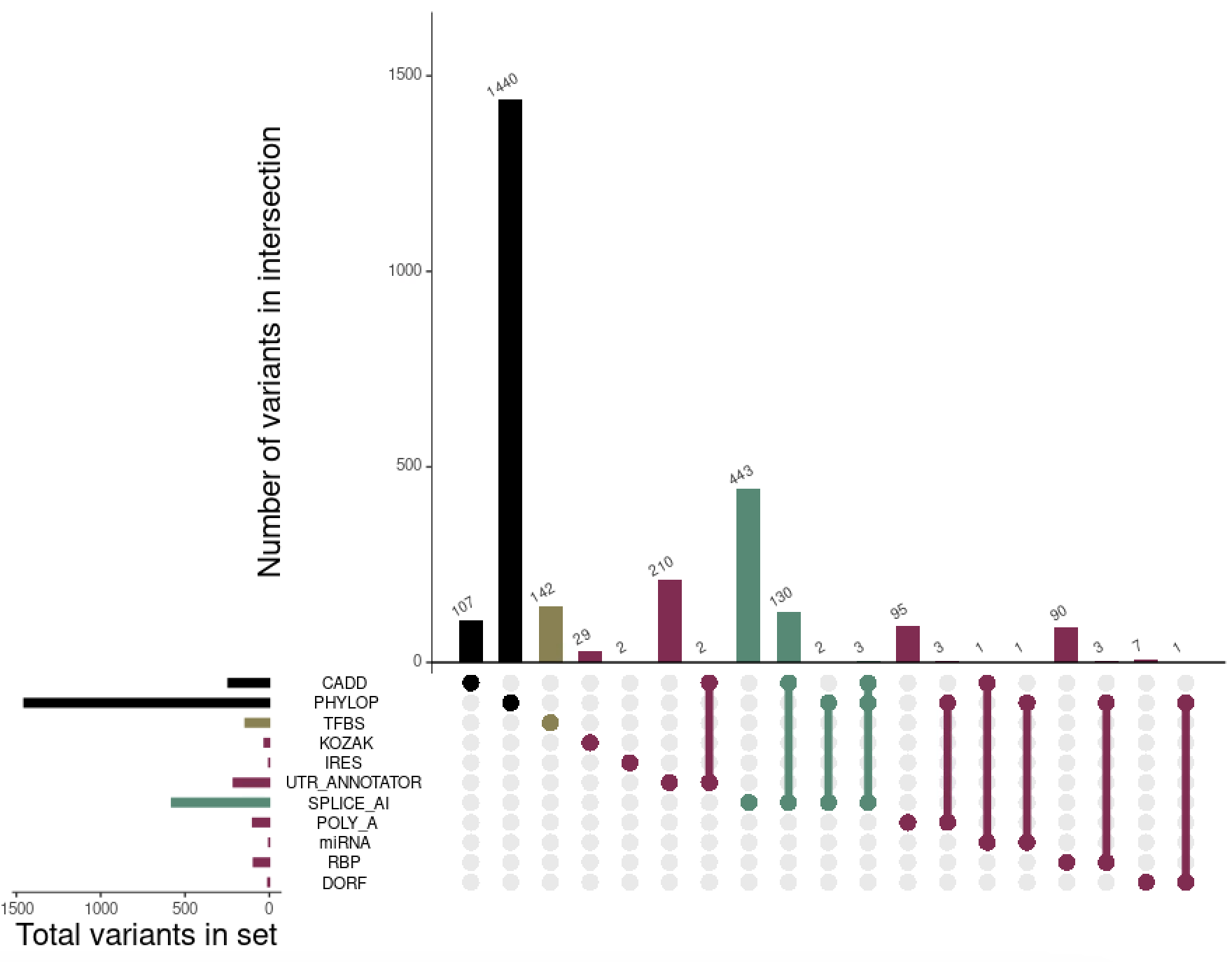
*

**Figure S5**: Annotated variants in the aggregated variant set used for burden testing. Universal variant annotations are shown in black, UTR specific variant annotations in raspberry, promoter specific variant annotations in mustard, and intronic / splicing variant annotations in teal. Vertical bars in the top panel denote the number of variants identified with specific region and variant annotations that are represented by the bar colour (regions), and in the upset plot below (variant annotations). The total number of DNVs with each variant annotation is shown by the horizontal bars to the left of the upset.


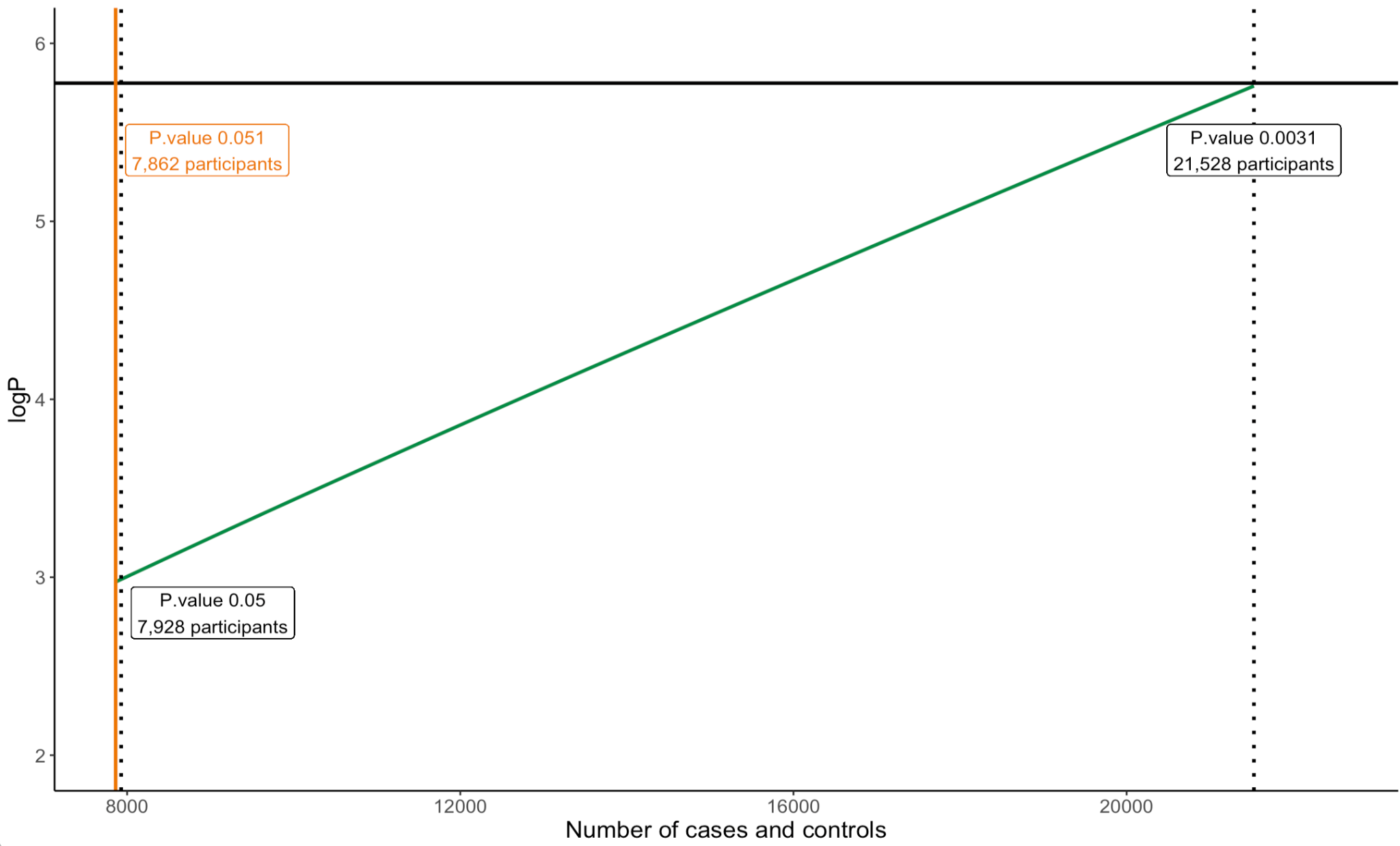


**Figure S6**: Estimated number of participants required to reach significance of *P<0.05* (left dotted line) and *P<0.0031* (right dotted line; Bonferroni adjusted threshold accounting for 16 tests) across all region and variant annotations. Observed *P*=0.051 shown as orange vertical line.
